# Evolution of Magnetic Symmetry through the BiFeO_3_–Ca_2_Fe_4/3_W_2/3_O_6_ Phase Diagram

**DOI:** 10.1021/acs.inorgchem.5c01516

**Published:** 2025-07-19

**Authors:** Catriona A. Crawford, T. Wesley Surta, Luke M. Daniels, Stanislav Savvin, Hongjun Niu, Jonathan Alaria, John B. Claridge, Matthew J. Rosseinsky

**Affiliations:** † Department of Chemistry, 4591 University of Liverpool, Crown Street, Liverpool L69 7ZD, U.K.; ‡ Leverhulme Centre for Functional Materials Design, The Materials Innovation Factory, 4591 University of Liverpool, 51 Oxford Street, Liverpool L7 3NY, U.K.; § 56053 Instituto de Nanociencia y Materiales de Aragón, CSIC−Universidad de Zaragoza, Facultad de Ciencias C/Pedro Cerbuna 12, Zaragoza, Spain and Institut Laue-Langevin, 71 avenue des Matyrs, CS 20156, 38042 Grenoble, Cedex 9, France

## Abstract

Perovskites offer vast flexibility in tuning subtle distortions in their structures through their innate ability to host a wide range of compositional combinations. Minor changes in composition can dramatically influence the properties observed through structural distortions such as octahedral tilting. In addition to understanding their properties, in magnetic materials, the magnetic structure is also tied to the nuclear structural distortions and can have more complex behavior with changing composition. In this work we report on the magnetic properties, and nuclear and magnetic structures of the solid solution (1 – *x*)­BiFeO_3_ – (*x*/2)­Ca_2_Fe_4/3_W_2/3_O_6_. With the exception of BiFeO_3_, all samples show a weak ferromagnetic behavior arising from spin canting. We find that despite only one structural phase transition occurring from *R*3*c* to *Pnma* in this solid solution, the magnetic phase diagram is far more complex, with four distinct magnetic phases occurring in the compositional range 0.1 < *x* < 1. Using a combination of neutron and X-ray diffraction, we find that a crossover between long and short Fe–O bond lengths and divergence of Fe–O–Fe bond angles with composition drive the changes in magnetic structure and can be correlated to the resulting magnetic properties.

## Graphical abstract

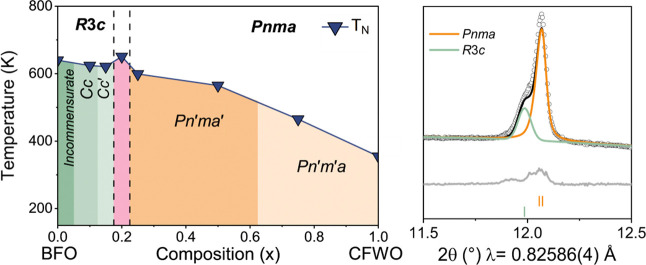

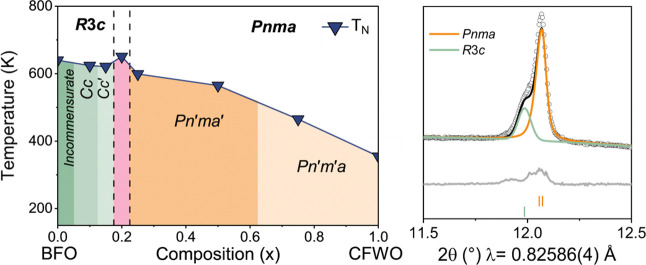

## Introduction

Designing multiferroics has been an area of high interest among solid state chemists and physicists for the last two decades due to the wide range of potential applications of materials possessing two or more degrees of ferroic order.^1–4^ Multiferroics with ferroelectric (FE) and ferromagnetic (FM) ordering offer the greatest interest for their technological implications; however, the inherent differences in the required electronic structures, typically d^0^ cations for FE properties or d^n^ cations for FM properties, make it difficult to incorporate both ferroic orders.^5^ Perovskites with the structural formula ABX_3_ afford the ability to incorporate different cations that may confer separate ferroic properties on the A and B sites, respectively, making them a typical avenue for designing new multiferroics. The archetype design of this can be seen in the well-known BiFeO_3_ (BFO) which crystallizes in space group *R*3*c* (Figure 1) and possesses FE polarization induced through the stereochemical nature of the Bi^3+^ (1.38 Å interpolated) lone pair effect, and Dzyaloshinskii-Moriya (D-M) interactions causing weak FM spin canting of the Fe^3+^ (0.645 Å) magnetic moments.^6–9^ However, there is an incommensurate cyclic modulation of this weak FM canted moment, resulting in an average antiferromagnetic (AFM) behavior.^10–12^ Additionally, the synthesis of phase-pure BFO is often plagued by the formation of thermodynamically stable impurity phases such as Bi_2_Fe_4_O_9_ or sillenite (Bi_12.5_Fe_0.5_O_19.5_),^13^ and due to the volatile nature of Bi^3+^, charge balancing oxygen vacancies are often present resulting in higher dielectric loss and electronic conductivity which causes difficulties in examining the FE properties due to the defects induced by Bi^3+^ volatilisation.

To exploit the weak FM order, the cycloidal modulation of spin canting must be disrupted or removed. One strategy to achieve this is through cation substitution on the A-site with a dopant cation that does not have a lone pair. The presence of a nonpolarizable cation on the A-site interrupts the long-range FE polarization, thereby influencing the D-M interactions underpinning the cycloid. Typical A-site doping strategies involve the substitution of Bi^3+^ for rare-earth or alkali-earth elements.^14^ When doped with rare-earth elements, the dopant ionic radius plays a pivotal role in the observed phase transitions. Larger A-site dopants, such as La^3+^ (1.36 Å),^15–17^ Nd^3+^ (1.27 Å)^18,19^ and Sm^3+^ (1.24 Å)^18,20^ are shown to have structural transformations from rhombohedral (*R*3*c*), to antipolar (*Pbam*), to nonpolar (*Pnma*) with increased doping levels. However, with smaller lanthanides such as Dy^3+^ (1.25 Å) and Ho^3+^ (1.23 Å) in which the compositions have the empirical formula Bi_1–*x*_Ln_*x*_FeO_3_, rather than the formation of an antipolar phase with doping, the increased size mismatch on the A site results in a monoclinic distortion to *Cc* which allows a rotation of the FE polarization axis and may have a weak FM canted moment allowed by symmetry.^21,22^ This monoclinic phase persists with increased doping until a region of phase coexistence with nonpolar *Pnma* occurs, for example, at 0.05 < *x* < 0.25 for Dy. When considering the alkali earth metals,^23,24^ substitution of larger Ba^2+^ (1.61 Å) and Sr^2+^ (1.58 Å) dopants results in a transformation to a pseudocubic or tetragonal nonpolar structure, crystallizing in *P*4/*mmm*. Substitution of the comparatively smaller Ca^2+^ (1.34 Å) has been shown to retain the *R*3*c* structure up to the nonpolar phase transition to *Pnma* (∼10% Ca substitution) without the formation of an antipolar *Pbam* phase.^23,25,26^ When greater than 60% Ca is present on the A site, the oxygen vacancies order to form a brownmillerite structure. A small magnetic hysteresis is observed upon 5% Ca substitution, meaning that, unlike the other alkali earth cations, Ca substitution can stabilize a weak FM moment while retaining a polar state. Further control of the magnetic properties is also possible through substitution on the B-site. BFO codoped with Ca^2+^ and a transition metal such as Ti^4+^ (0.605 Å), Mn^3+^ (0.645 Å) or Nb^5+^ (0.64 Å) all have suppressed the cycloidal magnetic ordering, but additionally show a larger compositional range of stability for the polar *R*3*c* phase and increased dopant levels before the eventual structural phase transition to nonpolar *Pnma* when compared to simply substitution Ca on the A site alone.^27–33^

By establishing the weak FM moment in bismuth-based compositions, it is possible to exploit these materials as multiferroics. Previous work has highlighted a solid solution between Bi­(Ti_3/8_Fe_2/8_Mg_3/8_)­O_3_ (BTFM) and CaTiO_3_ (CTO)^34–36^ which exhibits phase transitions from *R*3*c*, to *Pna*2_1_, to *Pnma,* with a region of phase coexistence between the two polar phases (*R*3*c* and *Pna*2_1_) known as a morphotropic phase boundary (MPB). All magnetically ordered compositions in the BTFM-CTO solid solution order above room temperature; however, at most, there is only 14 of the B-site occupied by a magnetic cation. In order to strengthen the magnetic interactions, which may result in a higher ordering temperature that is useful for multiferroic applications, we aim to design a solid solution that replicates the symmetry of the BFTM and CTO end members with a larger amount of magnetic cation (Fe^3+^) on the B-site. A solid solution between BFO and Ca_2_Fe_4/3_W_2/3_O_6_ ^37,38^ was chosen due to their respective octahedral tilting systems that matched those of BTFM (*a* ^–^ *a* ^–^ *a* ^–^) and CTO (*a* ^–^ *b* ^+^ *a* ^–^). Using Ca_2_Fe_4/3_W_2/3_O_6_ (CFWO) as an endmember also meant that the increased content of W^6+^ (0.60 Å) allowed the cations to charge balance and prevent the formation of oxygen vacancies ordering into a brownmillerite structure as in CaFeO_2.5_.

**Figure 1:**
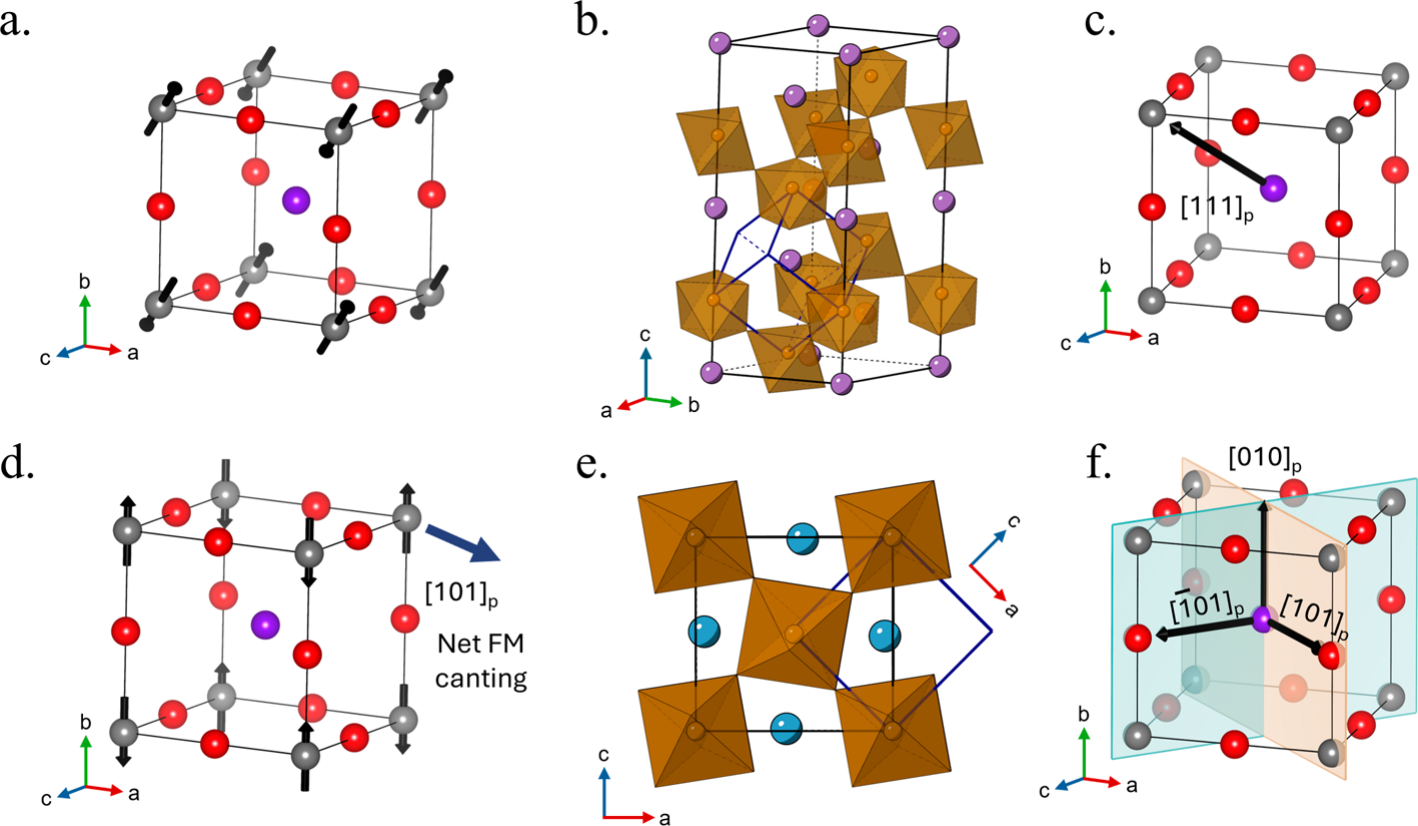
(a) Primitive cubic cell shows G-type AFM components to the magnetic ordering along [111]_p_ for BiFeO_3_. (b) BiFeO_3_ *R*3*c* unit cell (*a* ^–^ *a* ^–^ *a* ^–^) with the primitive cubic unit cell inset denoted by the blue axes, (c) Ferroelectric polarization along [111]_p_ for BiFeO_3_. (d) G-type AFM along [010]_p_ with the FM spin canting direction is shown along [101]_p_ for Ca_2_Fe_4/3_W_2/3_O_6_. (e.) Observed octahedral tilting in *Pnma* (*a* ^–^ *b* ^+^ *a* ^–^) with respect to the cubic unit cell is denoted by dashed lines. (f.) Possible ferroelectric polarization vectors derived from polar subgroups of *Pnma*, represented on a primitive cubic unit cell. The polar subgroups are as follows: *Pna*2_1_ ([101]_p_), *Pmn*2_1_ ([010]_p_), and *Pmc*2_1_ ([10–1]_p_). For (a), (c), (d), and (f), the gray atoms at the corners of the primitive cell are the B-site, red atoms along each edge are the oxygen atoms, and the purple atom in the center of the cell is the A-site. For (b) and (e), the brown octahedra denote FeO_6_ and the purple and blue A-site cations depict Bi (b) and Ca (e) respectively.

## Methods

### Synthesis

Powder samples were synthesized by solid state reaction along the (1 – *x*)­BiFeO_3_–(*x*/2)­Ca_2_Fe_4/3_W_2/3_O_6_ tie line, where *x* = 0.1, 0.15, 0.2, 0.25, 0.5, and 0.75. The samples were prepared by grinding and firing stoichiometric amounts of Fe_2_O_3_ (99.95% Alfa Aesar), WO_3_ (99.9% Sigma-Aldrich), CaCO_3_ (99% Sigma-Aldrich), and Bi_2_O_3_ (99.995% Alpha Aesar) in Al_2_O_3_ crucibles. A Fe_2_WO_6_ precursor was synthesized by firing the hand-ground Fe and W oxides at 1000 °C for 3 h with a heating and cooling rate of 5 °C/min. This was used as a precursor to suppress the formation of a thermodynamically stable CaWO_4_ impurity phase, which readily formed during synthesis. Ca_2_Fe_4/3_W_2/3_O_6_ was synthesized according to the literature,^37^ whereby the raw starting materials were hand ground with a pestle and mortar, then fired as loose powders at 800 °C for 1 h before cooling, regrinding, and further firing at 1200 °C with intermittent grinding after every 12 h. All intermediate compositions were milled in a high-speed Fritsch Pulverisette rotary mill in zirconia pots, with 10 mL of ethanol and seven 10 mm diameter zirconia balls for a total of 6 h in 25 min periods separated by 10 min intervals at rest. The powders were dried on a hot plate at 80 °C after milling. Dried powders were then mixed with a pestle and mortar and calcined as loose powders at a set temperature for 1 h as detailed in SI Table S1. The calcined powders were then ground and pressed into pellets using a 13 mm pellet die. The pellets were embedded under sacrificial calcined powder and fired at the set temperature for 12 h. Repeat 12-h firings with intermittent hand grinding were repeated until there were no changes to the powder X-ray diffraction (PXRD) pattern. BFO was synthesized by firing hand-ground Bi_2_O_3_ and Fe_2_O_3_ at 880 °C for 5 h.^13^ SI Table S1 details the temperatures used for calcining and annealing for each composition.

### Characterization

All materials were initially characterized in-house by PXRD on a Panalytical X’pert Pro instrument with a Co Kα_1_ (λ = 1.788960 Å) radiation source. High-resolution synchrotron PXRD data were collected on beamline I11, Diamond Light Source (λ = 0.82586 (4) Å) at room temperature using a MYTHEN detector. Birich samples were diluted with carbon to reduce absorption below 1 μR, and all samples were loaded into borosilicate capillaries. Powder neutron diffraction (PND) data were collected for CFWO and BFO at room temperature on the POWGEN time-of-flight powder diffractometer at Oak Ridge National Laboratory Spallation Neutron Source. For intermediate compositions, data were collected on the high-resolution powder diffractometer, D2B (λ = 1.5951(1) Å) at room temperature at the Institute Laue Langevin. Samples were loaded into cylindrical vanadium cans for all PND measurements. Data were analyzed by the Rietveld method using TOPAS Academic version 6, integrated with a jEdit text editor.^39^ A Chebyshev polynomial with 10 terms was used as a background function for all diffraction data. For PND data, a Thompson-Cox peak profile determined from a standard for each individual instrument was used. Peak broadening was fit by including Gaussian and Lorentzian strain and crystal size terms. For synchrotron PXRD data, the peak profiles were fit using the appropriate Stephens models to model any anisotropic peak broadening. Thermal displacement parameters for different atoms on the same site (i.e., Ca and Bi) were constrained to be equal. Lattice parameters were allowed to refine freely. Symmetry modes corresponding to atomic displacements with respect to the *Pm* 3̅ *m* perovskite aristotype was generated through ISODISTORT. The atomic coordinates were freely refined through these symmetry modes. The polyhedral distortion parameter for BO_6_ octahedra was calculated using eq 1. Where *a* is the cation coordination number, δ_*n*_ is the B–O bond length, and <δ> is the average bond length for that polyhedron.

$$\Delta =\frac{1}{a}\sum_{1}^{a}\left[{\left(\frac{\delta_{n}-<\delta >}{<\delta >}\right)}^{2}\right]$$ 1

Cation occupancies were set to the determined values from compositional analysis (see below) and were not refined. Identification of possible magnetic structures was investigated using ISODISTORT^40,41^ by identifying symmetry allowed magnetic subgroups and trialling each one by Rietveld refinement. Structural figures were made using a combination of VESTA^42^ and CrystalMaker software.^43^

Compositional analysis was performed by energy dispersive X-ray analysis (EDX) on a Hitachi S4800 SEM instrument, with data collection and analysis with Aztec software. The powdered sample was spread on a carbon tape with a silver electrodag paint applied to the edge of the carbon tape and aluminum stub. The sample was sputter-coated with carbon to prevent surface charging. The average composition for each sample was taken from at least 10 areas, and the standard deviation was taken as the error.

### Property Measurements

Magnetic measurements were performed on a Quantum Design SQUID-MPMS with the oven option for high-temperature measurements. Small sample pellets of roughly 20 mg were attached to MPMS oven heater sticks using alumina cement. DC magnetic susceptibility versus temperature (*M*(*T*)) measurements were recorded in the range between 298 and 750 K in zero-field cooled (ZFC) and 1 T field cooled (FC) modes. Magnetisation versus applied field was collected at room temperature in the range of −7 to 7 T at room temperature.

## Results and Discussion

### Synthesis and Structural Trends

A total of eight compositions were synthesized to establish the solid solution (1–*x*)­BiFeO_3_–(*x*/2)­Ca_2_Fe_4/3_W_2/3_O_6_, where *x* = 0, 0.1, 0.15, 0.2, 0.25, 0.5, 0.75, and 1. Compositional analysis by SEM-EDX confirmed the stoichiometry of each sample is provided in SI Table S2. PXRD data are shown in Figure 2a,b and highlight the change in symmetry across the solid solution. Figure 2b focuses on the (110)_c_ reflection, where c indicates the relation to the *Pm* 3̅ *m* aristotype peak which is split into two reflections when *x* < 0.2 illustrating the rhombohedral (*R*3*c*) elongation along [111]_c_. When *x* > 0.2, the peak splits into three reflections typical of an orthorhombic (*Pnma*) perovskite. At *x* = 0.2, the peaks are broader than the other compositions, and reflections from both *Pnma* and *R*3*c* symmetries can be observed, indicating a convolution of two phases. This can be clearly seen in the (110)_c_ peak in Figure 2b and the Rietveld refinement in Figure 2c. Phase coexistence is common at phase boundaries between symmetries that do not have a discrete, symmetry-allowed transition. The lattice parameters as a function of composition are shown in Figure 2d and show a decrease in unit cell parameters with reducing BFO content due to the decrease in ionic radii between Bi^3+^ and Ca^2+^. There is a linear change in the unit cell volume within the rhombohedral and orthorhombic regions, respectively, with a discontinuity in volume between these regions, further highlighting the first-order phase transition. Minor impurities, either scheelite (CaWO_4_) or sillenite (Bi_12.5_Fe_0.5_O_19.5_), existed in samples, but accounted for less than 3 wt % of the samples, as determined by Rietveld refinement, e.g., Figure 2c. Magnetic contributions from sillenite were not observable in any of the magnetic data.

**Figure 2:**
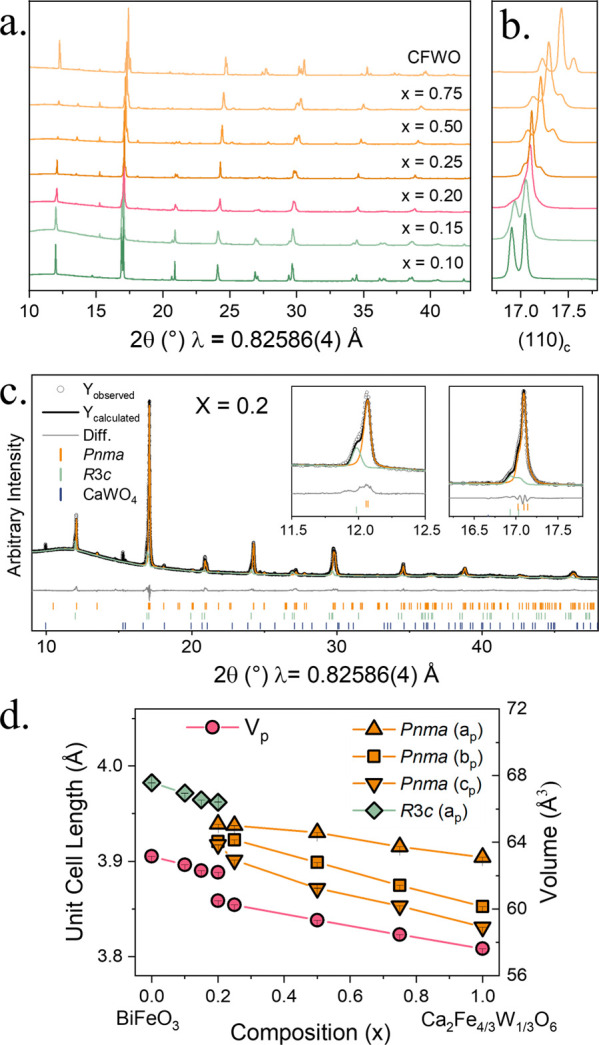
(a) Overlay of PXRD data collected on Beamline I11 at Diamond Light Source (λ = 0.82586(4) Å) showing the solid solution of (1–*x*)­BiFeO_3_–(*x*/2)­Ca_2_Fe_4/3_W_2/3_O_6_. (b) Selected 2θ range highlights the change in symmetry in the (110)_c_ peak from *R*3*c* (*x* = 0.1–0.2) to *Pnma* (*x* = 0.2–0.75 and CFWO). (c) Rietveld refinement of the structural model for *x* = 0.2 against PXRD data collected at Ill highlighted the presence of separate rhombohedral (green line) and orthorhombic (orange line) phases. The black *Y*_calc_ line represents the overall fit to the data which incorporates both *Pnma* and *R*3*c* structural models and the CaWO_4_ impurity phase. (d) Change in primitive cubic lattice parameters and volumes across the series.

The CFWO (*x* = 1) endmember has previously been reported to display either partial B-site order or cation disorder depending on the synthesis method.^37,38^ The large charge difference between Fe^3+^ and W^6+^ can result in a partial rocksalt type ordering of cations on the B-site with an Fe-rich (Fe = 73%, W = 27%) B′ site and Fe-poor (Fe = 60%, W = 40%) B″ site, crystallizing in *P*2_1_/*n* symmetry as determined by Rietveld refinement, when synthesized using a sol–gel precursor step prior to sintering.^38^ However, our refinement of the B-site occupancies resulted in an equal distribution over both sites with an Fe^3+^ occupancy of 66.7(2)% and W^6+^ occupancy of 33.3(2)%, which can be modeled by the higher symmetry *Pnma* space group and is concordant with the results seen using a purely solid state synthesis method by Ivanov^37^ which results in a very small magnetic moment that arises from weak FM spin canting and a disordered B-site. Structural details are provided in SI Table S3. Additional refinements in *P*2_1_/*n*, where the occupancies of the two B-sites were fixed to those reported in the rocksalt ordered structure, provided no improvement to the fit, and so the higher symmetry *Pnma* model was used from that point onward.

### Magnetic Property Measurements

Magnetization data were collected to understand the progression of properties through solid solution. Both endmembers have magnetic transitions above room temperature, so the magnetic susceptibility versus temperature, *M*(*T*), was collected for all compositions from 300 to 700 K and is shown in Figures S1a–f. The antiferromagnetic transition temperature (Néel temperature, *T*_N_) demonstrates a smooth change in this transition between endmembers (Figure 3d, Table S4), with a divergence from this trend at the mixed phase region (*x* = 0.20). The degree of orbital overlap between the Fe 3d orbitals determines the Fe–O–Fe superexchange interactions, and when these bond angles are close to linearity, the spin configuration is more AFM-like in nature, thereby increasing the magnetic ordering temperature. All intermediate compositions behave similarly to CFWO, whereby a divergence of the field-cooled (FC) and zero-field-cooled (ZFC) measurements is observed below *T*_N_ (Figure S1a). The ZFC data for 0.1 < *x* < 1 show AFM behavior, whereas the FC data show FM behavior with a small magnetic moment, indicative of a noncollinear magnetic structure with uncompensated FM spin canting. The very small magnetic moment is concurrent with the magnetic results observed in the literature reporting this synthesis method and weak ferromagnetic spin canting, whereas other reports of ferrimagnetism from an ordered B-site have magnetic susceptibilities that are of an order of magnitude.^37,38^ The inverse susceptibility data were fit by using the Curie–Weiss law in the paramagnetic region above *T*_N_. For CFWO, a linear fit yields a θ_CW_ = 21 K, indicating a small net FM moment, and a μ_eff_ = 5.62 μ_B_, within the expected range for a 3d^5^ transition metal (μ_observed_ = 5.6–6.1 μ_B_).^44^

**Figure 3:**
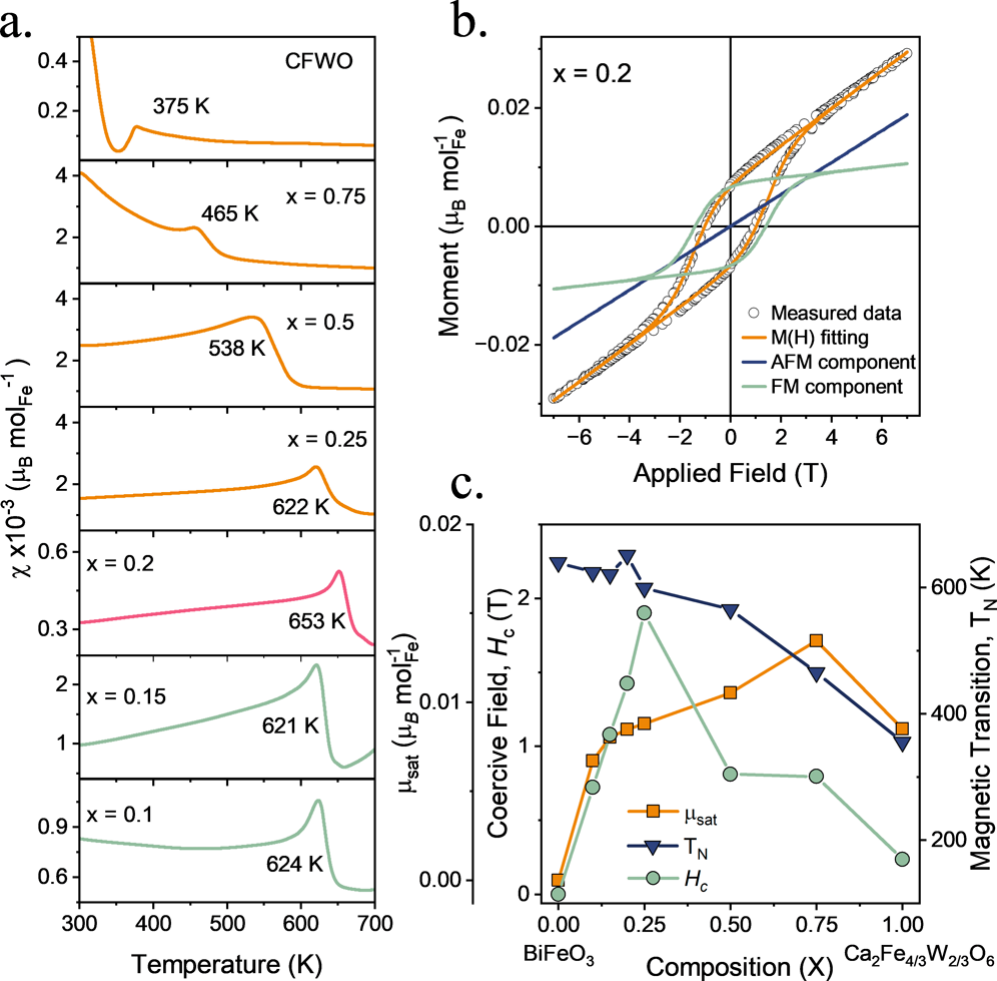
(a) ZFC *M*(*T*) component for all compositions showing the change in the AFM *T*_N_ with *x*. (b) *M*(*H*) loop fitting was used to extract the μ_sat_ and coercive field for each composition. (c) Extracted parameters were extracted from *M*(*T*) and *M*(*H*) data. Blue triangles denote *T*_N_, orange squares denote saturation magnetization, and green circles are the coercive field.

The BFO–CFWO system was investigated with the goal of tailoring a compound which was magnetically ordered at room temperature, and so the magnetic susceptibility versus applied field (*M*(*H*)) was measured for all compounds and is provided in Figure S2a,b. For compositions where *x* = 0.1–1, the susceptibility does not fully saturate due to the predominant AFM interactions and the field being below the spin flop transition; however, all show FM hysteresis. For CFWO, the observed magnetic susceptibility measured is far smaller than that reported in the ferrimagnetic, B-site ordered CFWO,^38^ and is more in line with the B-site disordered structure,^37^ giving further evidence of the origin of the weak ferromagnetism being due to the spin canting. Due to the existence of AFM and FM components, these data were modeled with a hyperbolic tangent function:

$$M\left(H\right)=\underset{i}{\sum }m_{i}\left(H\right)$$ 2

where

$$m_{i}=\alpha \tan h\left(\frac{H-b}{c}\right)+dH$$

and represents a single magnetic component.^35^ This modeling allowed the extraction of key information from the measurements, such as the coercive field (*H_c_*) and saturation magnetization (μ_sat_). Here α = μ_sat_, *b* = *H_c_*, *c* is a fitting parameter describing the ‘squareness’ of the ferromagnetic hysteresis loop, and *d* is a linear term describing the para-, dia-, and antiferromagnetic contributions to the individual magnetic moments. By splitting the function into a hyperbolic tangent and a linear term, as shown in Figure 3b, it is possible to separate the components arising from weak FM and AFM ordering.

The extracted values for μ_sat_ and *H_c_* are shown in Figure 3c. As nonmagnetic W^6+^ is introduced on the B-site, the magnetic superexchange interactions and hence the cycloidal nature of the spin canting structure are disrupted. This results in the observation of the weak FM component shown by the increase in μ_sat_ from BFO. As the amount of Fe^3+^ on the B-site decreases across the solid solution, an increase in the μ_sat_ in the intermediate compositions is observed, which is likely related to changes in octahedral tilting throughout the series. There is a decrease in *T*_N_ with decreasing *x*, which will be highly correlated to the change in orbital overlap, influencing the Fe–O–Fe superexchange interactions, but also is related to the reduction of the percolation of these interactions over a long range without being interrupted by W^6+^. A higher percolation of the superexchange network will result in a higher magnetic ordering temperature. The large coercive field strength (*H*_*c*_) peaks at *x* = 0.75, and describes the energy associated with reorientating the magnetization, and an understanding of the structural distortions that are the driving force behind this observation is required to make further conclusions.

### Nuclear and Magnetic Structural Analysis

The trends in the magnetic properties followed the variation in the amount of Fe^3+^ on the B-site; however, the *x* = 0.2 composition at the phase boundary had both an outlying *T*_N_ and a large *H*_*c*_. These features made this sample of particular interest, prompting the exploration of the causes of these changes. In the PXRD data, reflections corresponding to both *R*3*c* and *Pnma* symmetry were identified, and a Rietveld refinement of the nuclear structure (Figure 2c) confirms the presence of these two phases. The weight fractions refined to 86.7 and 13.3% for the *Pnma* and *R*3*c* phases, respectively, indicating that *x* = 0.20 is closer to the orthorhombic symmetry side of the mixed phase region.

Neutron diffraction was pursued to study the chemical origin of the magnetic properties across the series, by enabling more accurate oxygen positions to be established, which in turn allows trends in the bond angles and octahedral tilting to be drawn; and additionally, determining the magnetic structures. Understanding how the magnetic symmetry changes across the solid solution plays a vital role in deciphering the magnetic properties; however, the coexistence of two phases makes determining the magnetic symmetries challenging due to the presence of overlapping reflections. To allow the accurate determination of the magnetic structures at the phase boundary, *x* = 0.2, a methodical approach to determining the magnetic symmetry across the full solid solution was undertaken. For all compositions, the magnetic reflections in the neutron diffraction data could be indexed with a *k* vector of *k* = [0, 0, 0], meaning that there was no superstructure required to encompass the AFM ordering. On investigating the magnetic symmetry by Rietveld refinement, multiple changes to the magnetic structure were observed across the series. For each composition, symmetry allowed magnetic subgroups were identified using ISODISTORT with respect to their nuclear structures, and the quality of fit was trialled by Rietveld refinement and is shown in Figures 4 and 5 and is reported in Table S5. The Rietveld fits and structural information for each composition can be found in the Supporting Information, Tables S6–S11, and Figures S3–S8. Figure 6 shows the stacked plot of the fit to the most intense magnetic peak for compositions 0.1 < *x* < 0.75.

**Figure 4:**
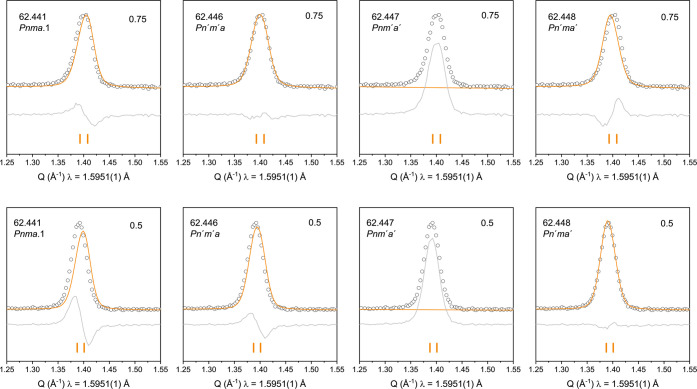
Rietveld fit of the most intense magnetic peak in the NPD data for *x* = 0.75 (top row) and *x* = 0.5 (bottom row) highlighting any intensity mismatch between different magnetic symmetries trialled.

**Figure 5:**
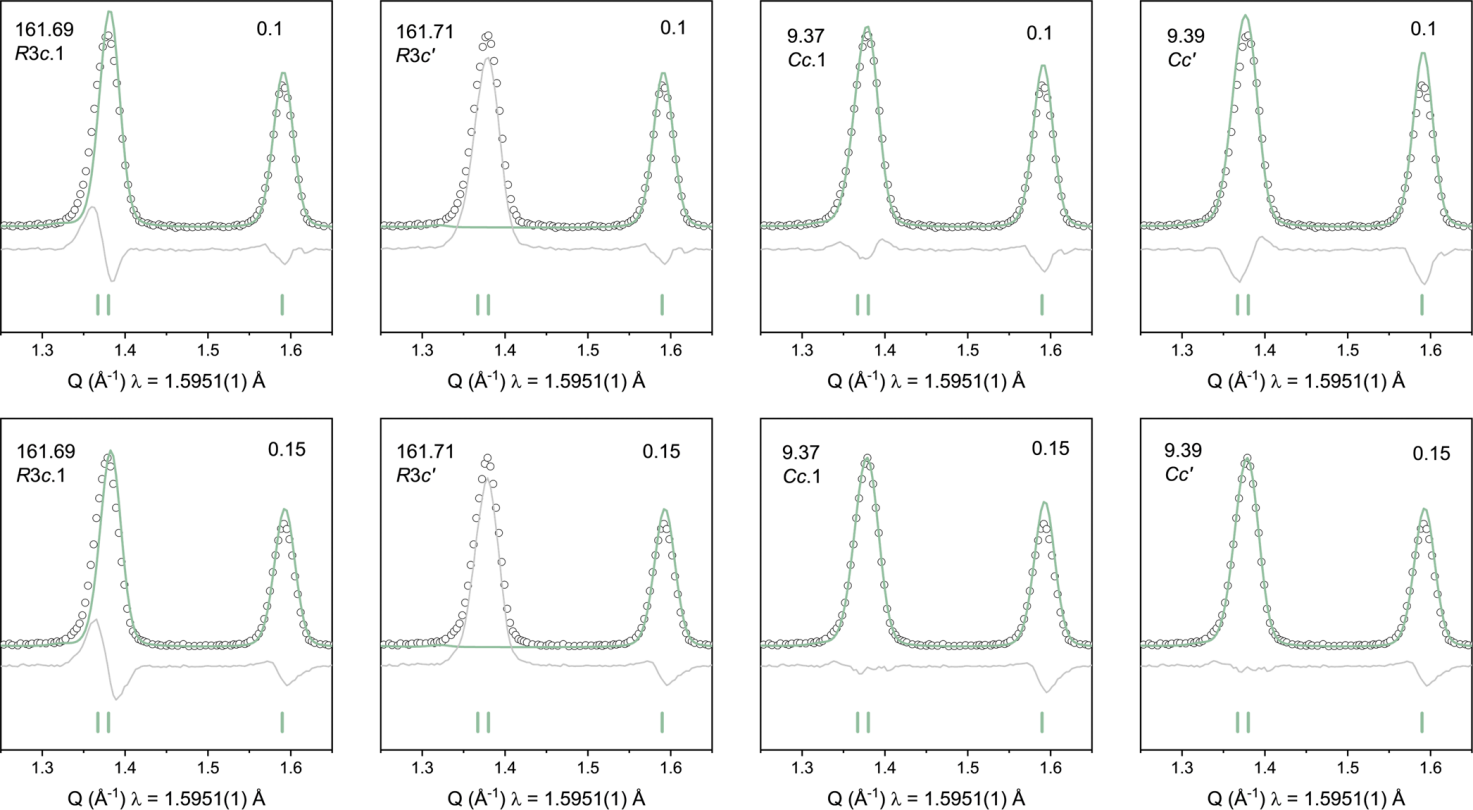
Rietveld fit of the most intense magnetic peak in the NPD data for *x* = 0.1 (top row) and *x* = 0.15 (bottom row) highlighting any intensity mismatch between different magnetic symmetries trialled.

**Figure 6:**
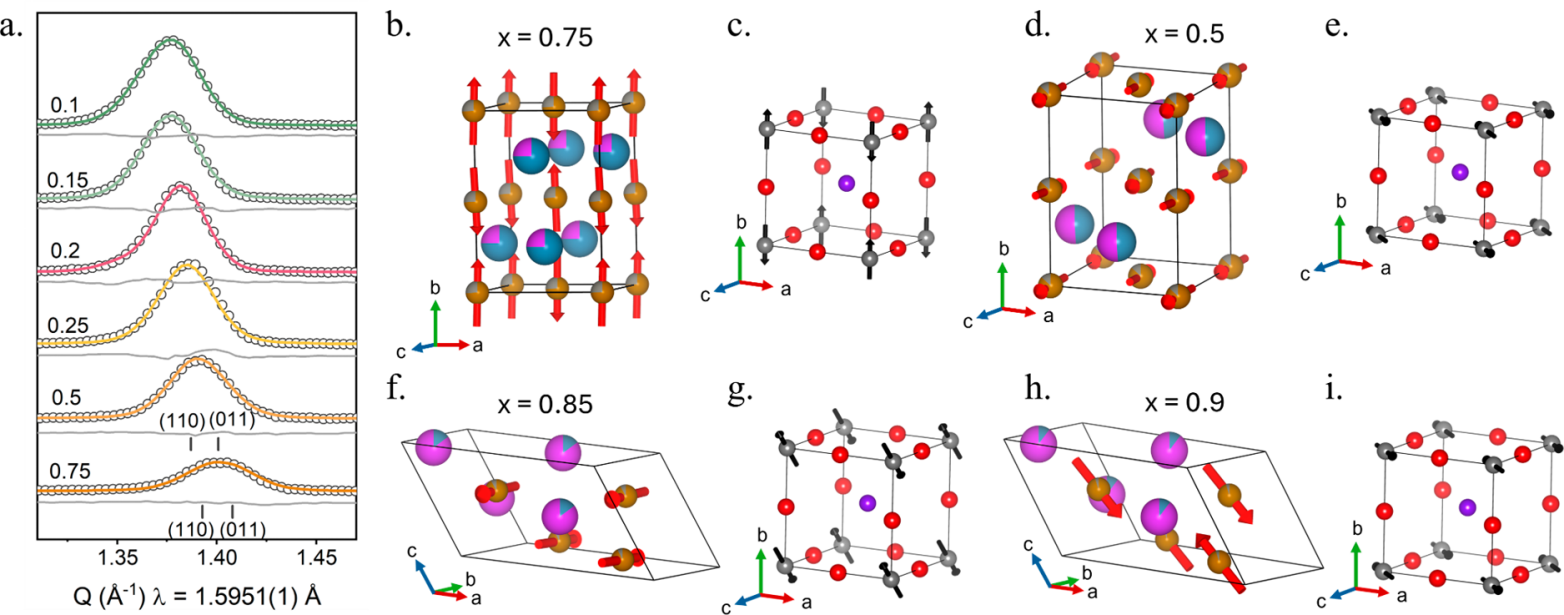
(a) Rietveld fit of the strongest magnetic reflections of compositions *x* = 0.75–0.1. The (110) and (011) are shown for *x* = 0.75 and 0.5 to highlight the subtle change in intensity between the two different magnetic structures, determining the change in direction of the magnetic vectors. The magnetic structure and direction of magnetic vector with respect to the cubic parent are shown for (b, c) *x* = 0.75 [010]_*c*_, (d, e) *x* = 0.5[101]_*c*_, (f, g) *x* = 0.15 [011]_*c*_, and (h, i) *x* = 0.1 [101]_*c*_.

For the orthorhombic compositions at *x* = 1 and 0.75, the magnetic symmetry *Pn′m′a* (mΓ_2_^+^ BNS number 62.446) was observed. Previous studies on the magnetic structure of CFWO had determined a ferrimagnetic structure, which was concurrent with the partial B-site order observed when synthesized through sol–gel methods.^38^ However, as we do not observe any B-site order and the determined magnetic space group, *Pn′m′a*, has weak FM spin canting allowed by symmetry, no further symmetry lowering was required. These structures show a G-type AFM arrangement ordered *b* ([010]_*c*_) with a weak-FM component allowed along *c* ([101]_*c*_) due to canting of the spins (Figure 6b). For the refinements of both compositions, the weak-FM moment along *c* was set equal to 0 and not refined, as it was too small to have any observed intensity that could be modeled. The fit to the neutron diffraction data and the final refined magnetic and nuclear structure (for *x* = 0.75) can be seen in Figure S3 and Table S6. There is an additional AFM component along *a* which manifests as a small, compensated canting of the magnetic moments between neighboring octahedra in that direction. Figure S9 shows the differences in the weaker AFM component and the wFM component for *x* = 0.75 (*Pn′m′a*) and 0.5 (*Pn′ma′*), which have different magnetic structures (a small arbitrary value of wFM component used for visualization in figures). For *x* = 0.5, 0.25, and 0.2, the magnetic symmetry was determined to be *Pn′ma′* (mΓ_4_^+^, BNS number 62.448). This symmetry has its G-type AFM ordering rotated 90° compared *Pn′m′a* (mΓ_2_^+^) with the AFM moments to be aligned along *c* ([101]_*c*_) as shown in Figure 6d and a weak-FM component allowed due to spin canting occurring along *b* ([010]_c_) which was also set to 0 for the refinement. A figure showing the refinement of *x* = 0.25 in both *Pn′ma′* and *Pn′m′a* is provided in Figure S10. To ensure that this change in magnetic symmetry was present, and there was no possibility of modeling the magnetic structures for all orthorhombic compositions in the same magnetic space group, a series of models were trialled for both *x* = 0.75 and 0.5. These models simulated the diffraction patterns for magnetic structures produced using symmetry mode refinements generated using ISODISTORT, with fixed mode amplitudes corresponding to changes in the magnetic structures. This included either increased canting or a change in direction of the magnetic vector for both *Pn′ma′* and *Pn′m′a*. For both compositions, increasing the AFM canting or changing the direction of the magnetic vector in both magnetic space groups resulted in fits to the data of reduced quality. Increasing the weak FM canting made no difference to the fit quality, and the different models and refinement outcomes can be seen in Figures S11 and S12.

On the rhombohedral side of the phase diagram, BFO (*x* = 0) has a G-type AFM ordering with moments oriented along the Bi^3+^ polarization axis, *c* ([111]_*c*_). However, a magnetic superstructure with an incommensurate helical canting of the spins orthogonal to [111]_*c*_ results from Dzyaloshinskii-Moriya interactions between spins. The cycloidal nature of this spin canting means that, on average over many unit cells, the weak-FM is canceled out, leaving only the contributions from the AFM moments observable in property measurements. Substitution on the A-site with a nonpolarizable cation, as discussed in the Introduction, has been shown to interrupt the DM interactions and generate a weak-FM moment, which is evidenced in this work by the hysteresis in the *M*(*H*) measurements upon substitution. The magnetic subgroups of the parent space group *R*3*c* do not have allowed weak-FM components, and their use to fit neutron data resulted in an intensity mismatch on the magnetic reflection at *Q* = 1.39 Å^–1^ (Figure 5). Therefore, lower symmetry magnetic space groups were trialled, which allow a rotation of the direction of the magnetic moments.

For *x* = 0.1, applying a group theoretical approach revealed two possible magnetic structures: *Cc* (mΓ_3_, BNS number 9.37) and in *Cc′* (mΓ_3_, BNS number 9.39). Magnetic refinements in *Cc*′ resulted in poorer quality fits to the shape of the magnetic peak shown in Figure 5 (top right), and the magnetic structure could be more appropriately modeled using *Cc* symmetry (Figure 6h). In this symmetry, the AFM moments lie along *c* ([101]*_c_*) and have weak-FM canting of the spins along *b* ([011]_*c*_). This *Cc* magnetic structure has been observed in BiFeO_3_ substituted with other small A-site cations such as Ho^3+^ and Dy^3+^ and in epitaxially strained thin films.^21,22,45^ At *x* = 0.15, the quality of the fit in both *Cc* and *Cc*′ models appeared to be similar, with a slightly lower *R*_wp_ found for the *Cc*′ model (Table S5) and a refined magnitude of 3.78 (2) μ_B_. However, on closer inspection of the magnitude of the magnetic moments, the value for the *Cc* model (4.28 (8) μ_B_) was considerably larger than that for the rest of the series (around 3.7 μ_B_). While a value of 5 μ_B_ would be expected for a high-spin d^5^ cation, the value measured at 300 K would likely be lower due to thermal fluctuations. With this in mind, *Cc*′ symmetry is the most appropriate model to describe the experimental data for *x* = 0.15 and 0.2. Here, the AFM ordering is parallel to the *b* ([011]_*c*_) axis, and the canted moment is aligned along the [101] of the *Cc* unit cell ([100]_i_) as shown in Figure 6f. Similarly to the orthorhombic compositions, additional magnetic models with increased canting or changes in magnetic vector direction were trialled for both *x* = 0.1 and 0.15, and the results are provided in Figures SI13 and SI14. In *x* = 0.1, increasing the canting or changing the direction of the magnetic vector both reduce the quality of the fit to the experimental data in both *Cc* and *Cc*′. In *x* = 0.15, while comparable fits are obtained in both magnetic symmetries, the refined moment of 3.78 (2) μ_B_ from the *Cc*′ model agrees well with the rest of the composition series, compared to the value 4.28 (8) μ_B_ from the model in *Cc,* thus confirming the former as the most appropriate symmetry. Furthermore, increasing the canting angle or changing the magnetic vector direction in both models also results in poorer fits.

Despite the simplicity of the nuclear structure through the phase diagram, which goes from *R*3*c* to *Pnma* with a mixed phase region at the phase boundary (*x* = 0.2), the materials present a much richer magnetic phase diagram. Trialling variations in canting and magnetic vector directions for the different magnetic models of *x* = 0.75, 0.5, 0.15, and 0.1 (Figures S11–S14) did not lead to any improvements of the freely refined models. We further conclude that it was not possible to accurately describe the magnetic structure with a single magnetic space group (per nuclear structure region), even with increased canting or changing the direction of the magnetic vectors, and therefore changes in magnetic symmetry are required within both the orthorhombic and rhombohedral regimes of that compositional space. Five different magnetic symmetries are observed with subtle changes that have an impact on the resultant magnetic properties. Using the information extracted from Rietveld refinements for each composition, it is possible to understand these changes, which are shown in Figures 6 and 7a.

**Figure 7:**
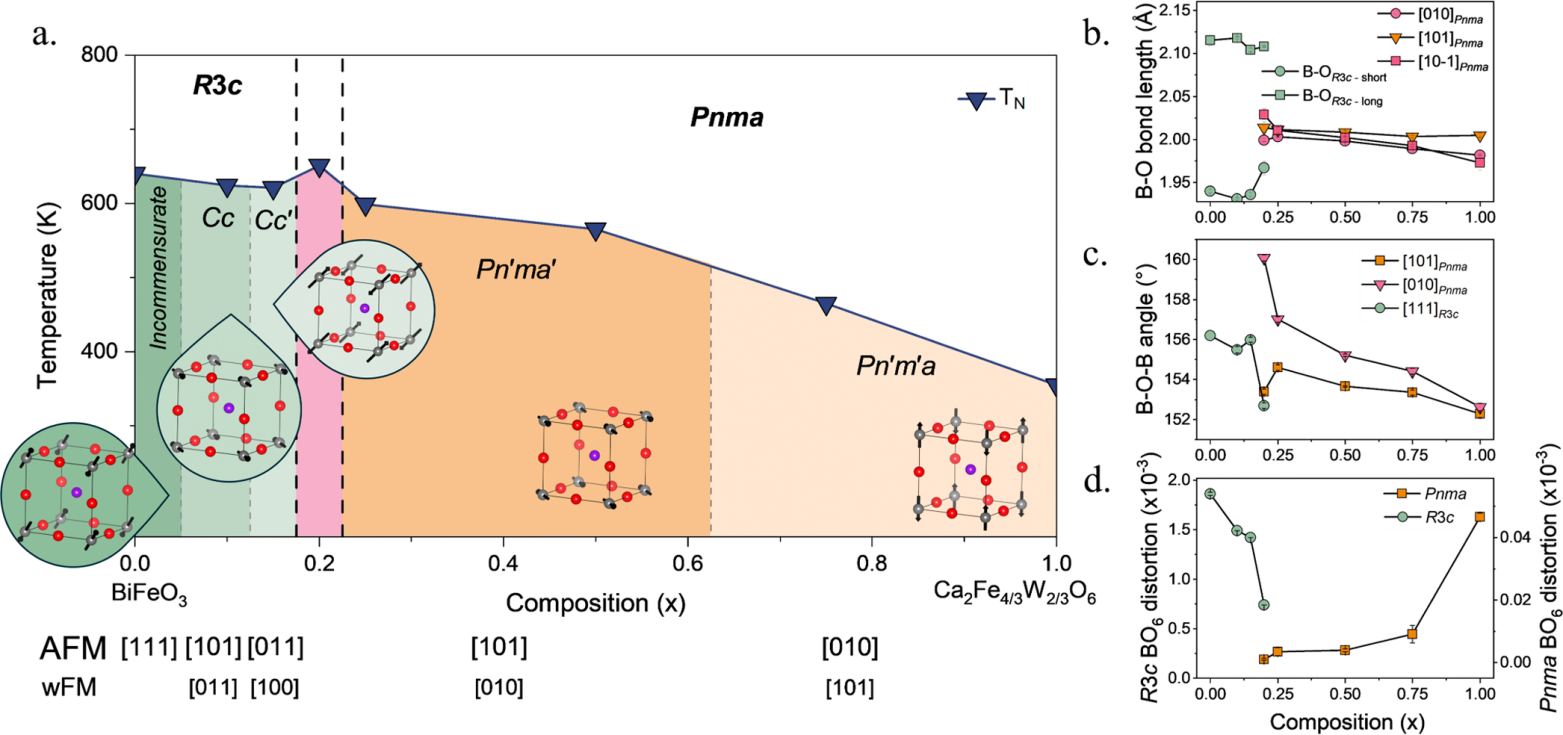
(a) (1–*x*)­BiFeO_3_–(*x*/2)­Ca_2_Fe_4/3_W_2/3_O_6_ phase diagram highlights the change in nuclear and magnetic symmetry. For phase transitions where the exact composition of transition is not known a dashed line is used to provide rough phase boundaries. Blue triangles denote the *T*_N_ for each composition, and the direction of the AFM moments with respect to the primitive cubic cell are shown for each symmetry. The vectors for the AFM moment and weak FM moment w.r.t. the cubic cell are located below the phase diagram. (b–d) Variation in (b) B–O bond length, (c) B–O–B bond angle and (d) polyhedral distortion parameters across the (1–*x*)­BiFeO_3_–(*x*/2)­Ca_2_Fe_4/3_W_2/3_O_6_ solid solution.

The magnetic properties in AMO_3_ (where M is a 3d transition metal with unpaired electrons) perovskite oxides are highly dependent on the B-site *3*d and oxygen 2p orbital overlap, which in turn depend on the BO_6_ bonding environments and the connectivity of polyhedra, i.e., the octahedral tilting. *Pnma* symmetry has two identical, out-of-phase octahedral tilts ([101] and [ 101̅ ]) and one unique in phase tilt ([010]), represented as *a* ^–^ *b* ^+^ *a* ^–^ in Glazer notation. In CFWO (*x* = 1), the B–O–B bond angles, Figure 7c, along the [101] and [010] are similar in magnitude (152.28(2) and 152.636(2)° respectively), however the B–O bond length along [101] (2.005(2) Å) is larger than the other B–O bonds along [10–1] (1.973(9) Å) and [010] (1.982(2) Å) directions shown in Figure 7b. This long bond along [101] persists with increasing Bi^3+^ and Fe^3+^ content until *x* = 0.5, by which point the in-plane disparity between the [101] and [ 101̅ ] bond lengths are much smaller, and all B–O bond lengths become almost equal (approximately 2.004 Å), and a change in the magnetic symmetry is observed. These changes to the bond lengths are the driving force for the change in magnetic symmetry from *Pn′m′a* to *Pn′ma′*.

In RFeO_3_ perovskites (R = rare earth) with the *Pnma* structure, the magnetic easy axis (the axis of highest symmetry within an octahedron^46^) and therefore magnetic moments are driven by variations in the bond lengths. In this case, the magnetic easy axis lies between the largest and smallest bond lengths, the [101] and [ 101̅ ], resulting in moments aligning along the *a* or *c* unit cell axis.^47^ This additionally avoids any energetically unfavorable alignment of the moments toward each other along the [010] bond lengths. When R is a magnetic cation, a further spin reorientation transition occurs where the magnetic moments rotate to align along *b* (i.e., along the Fe–O–Fe bonds) and is driven by R-Fe interactions.^46,48–50^ However, the case may differ when other (non)­magnetic cations are introduced on the B-site, for example, in Sr_3_Fe_2_TeO_9_, Sr_3_Fe_2_WO_9_, Sr_3_Fe_2_ReO_9_, and R_3_Fe_2_MoO_9_ (R = Nd, Pr, Ce, La), all have their magnetic moments directed along Fe–O–Fe bond lengths.^51–55^ This suggests that with significant disruption of the magnetic superexchange interactions through occupational disorder, the orientation of the magnetic moments may have more freedom to rotate.

In this work, the compositions, *x* = 1 and 0.75, that have the greatest difference in bond lengths within the *a*/*c* plane and the smallest bond angles, have their magnetic moments aligned along *b*, along an Fe–O–Fe pathway as observed in the A_3_Fe_2_MO_9_ perovskites mentioned above. Whereas compositions, *x* = 0.25 and 0.5, have little bond length variation but a more linear Fe–O–Fe [010] bond angle and have their moments aligned along *c*, like the RFeO_3_ perovskites. This result reveals an interesting observation that in B-site doped orthoferrite perovskites, the magnetic easy axis direction is affected by not only variations in bond lengths but also the bond angle variations arising from octahedral tilting. This can be seen in Figure 7b,c, showing the interplay between the bond length and bond angle variation on the magnetic structure. In compositions where *x* = 0.25 and 0.5, the difference in Fe–O bond lengths is too small to drive the magnetic moment orientation alone. However, the bond angles along the *b*-axis are significantly larger and closer to linear than in the *ac* plane, which drives the rotation of the AFM ordering direction into the *ac* plane ([101]) to avoid the energetically unfavorable alignment of the moments along *b*. In compositions where *x* = 0.75 and 1, rather than the magnetic easy axis being driven by variation in bond lengths (as seen in the RFeO_3_ perovskites), the decreasing [010] bond angle and variation in bond angles with increasing *x* result in a lower energy barrier to aligning the moments along *b* compared to those with *x* = 0.5 and 0.25. The smaller bond angles along [010] mean that the magnetic spins will be canted further away from linearity, relieving the magnetic frustration. This rotation of the magnetic easy axis is likely enabled by the increased disruption to the magnetic superexchange with increasing compositional disorder as the amount of W increases.

The variation in *T*_N_ can also be attributed to the decrease in octahedral tilting and increase in B–O–B bond angle with decreasing *x* values (Figure 7c), and not just to an increased Fe^3+^ content. The expansion of B–O–B bond angles closer to linear (180°) along the possible superexchange pathways results in a far greater degree of orbital overlap between the Fe 3d and the O 2p orbitals and therefore a more AFM-like nature in the alignment of the spins, leading to a higher transition temperature. This can be seen across the solid solution with decreasing *x*, except for *x* = 0.2. This composition has the most linear bond angle of 160.01(2)° along [010] in the orthorhombic phase compared to any other composition in the series. This linear B–O–B angle is concurrent with the anomalously high *T*_N_. Similar trends have been well studied in other systems by varying the A-site radii in LnMO_3_ materials (where Ln is a lanthanide and M = Cr^3+^, Mn^3^, or Fe^3+^).^56^

With an understanding of the magnetic symmetries and structural trends on the orthorhombic side of the phase diagram, the rhombohedral side was examined. In the rhombohedral compositions, as *x* decreases, there is an increase in the B–O–B bond angle and a divergence in the long and short B–O bond lengths. However, the change to the magnetic structure between *x* = 0, 0.1, and 0.15 cannot be explained by these structural distortions as done in the orthorhombic region. Since there are no structural indicators that clearly reveal these precipitous changes in symmetry, an electronic argument must be made, stemming from the introduction of small amounts of W^6+^ (d^0^) onto the B-site. The d^0^ orbitals participate in hybridization with the O 2p orbitals, which may induce W^6+^ (and O) displacements on a local scale, disrupting the magnetic superexchange by increasing the magnetic anisotropy and connectivity of the B-site polyhedra throughout the average structure, and may change the nature of the Fe–O–Fe superexchange. It is possible that the cycloidal spin canting is still present but can only permeate through the structure on a much shorter scale, compared to BFO, before being interrupted by W^6+^, which may be an underlying reason for the narrowing of the *M*(*H*) loop between 0.15 and 0.1.

Looking across the whole solid solution (Figure 7a), it is clear to see how the subtle structural changes affect the resulting magnetic structure and properties. The polyhedral distortion parameter, Δ, was additionally calculated for BO_6_ across the series (Figure 7d). Where there is compositional phase coexistence, the structures typically encompass a greater intrinsic strain, as two phases compete for formation. At *x* = 0.2, Δ is the smallest in both orthorhombic and rhombohedral structures. This may seem counterintuitive; however, in this case it indicates that as more chemical pressure is placed on a certain structure from changing the tolerance factor or influencing the orbital hybridization, the resulting structural distortions that occur (bond lengths and octahedral tilting) push the BO_6_ octahedra to the extremes within that polyhedral connectivity within that tilt system prior to the phase transition. In this case, as both phase regimes close in on *x* = 0.2, the change in composition pushes the polyhedra to become less distorted as the bond length difference reduces in magnitude to ensure that the appropriate A-site coordination can occur. The bond angles in the orthorhombic (primary) phase are also far closer to 180°, particularly along *b*, resulting in a far smaller degree of octahedral tilting. This increases the resulting orbital overlap along the AFM chains, resulting in a higher magnetic ordering temperature, and greatly increased resilience to switching the sign of the magnetic order under an opposing field, meaning a larger *H*_*c*_.

## Conclusions

We have synthesized various compositions across the (1–*x*)­BiFeO_3_–(*x*/2)­Ca_2_Fe_4/3_W_2/3_O_6_ solid solution using a solid-state synthesis method aided by ball milling. The nuclear structure changes from a polar, rhombohedral phase to a nonpolar orthorhombic phase that transitions through a region of phase coexistence at *x* = 0.2. Despite the commonly occurring structural evolution, the magnetic phase diagram is more complex, with multiple changes to the magnetic structure and rotation of the magnetic moments. Through Rietveld refinement on PND and PXRD data, we justify changes to the magnetic structure by extracting specific structural parameters and correlating them to the change in magnetic properties. The change in the orthorhombic magnetic structures (0.25 < *x* < 1) is driven by bond angle variations and a reduction in Fe–O–Fe connectivity with increasing W content. In the rhombohedral magnetic phases (*x* < 0.2), we conclude that the change in magnetic symmetry is electronically driven by the incorporation of a nonmagnetic d^0^ cation (with increasing *x* values) on the B-site, disrupting the magnetic superexchange and helical order observed in BFO. The mixed phase composition (*x* = 0.2) had the highest *T*_N_ due to the bond angles along the superexchange pathways that were closer to linear than all other compositions. All compositions where *x* > 0 showed magnetic hysteresis in the *M*(*H*) loops, confirming the disruption of the BFO helical ordering and the possibility of exploiting the weak FM moment. This work uncovers a rich magnetic phase diagram, and while there are only two nuclear structures in this phase diagram, there are five distinct magnetic structures which highlights how the subtle tuning of composition has an effect beyond that of just a change in crystallographic symmetry and how subtle structural changes can have profound effects on the magnetic symmetry.

## Supplementary Material



## Notes

Additional details for experimental work, compositional analysis, magnetic property data, details of structural analysis for all compositions, and trialled magnetic structures (PDF)
