# Supplementary material for: Evolution of Magnetic Symmetry through the BiFeO3–Ca2Fe4/3W2/3O6 Phase Diagram
Source: Inorg Chem. 2025 Jul 19;64(30):15413–23. doi: 10.1021/acs.inorgchem.5c01516 (PMC12326346; doi:10.1021/acs.inorgchem.5c01516)

Supporting Information

Evolution of Magnetic Symmetry Through the $BiFeO_3$ - $Ca_2Fe_{4/3}W_{2/3}O_6$ Phase Diagram

Catriona A. Crawford[a], T. Wesley Surta[a], Luke M. Daniels[a], Stanislav Savvin[c], Hongjun Niu[a], Jonathan Alaria[a], John B. Claridge[ab], Matthew J. Rosseinsky[ab].*

[a] Department of Chemistry, University of Liverpool, Crown Street, Liverpool, L69 7ZD, UK

[b] Leverhulme Centre for Functional Materials Design, The Materials Innovation Factory, University of Liverpool, 51 Oxford Street, Liverpool, L7 3NY, UK

[c] Institut Laue-Langevin, 71 avenue des Matyrs, CS 20156, 38042 Grenoble cedex 9, France

*Email: m.j.rosseinsky@liverpool.ac.uk

Table S1. Firing temperatures for different compositions in the (1-x)$BiFeO_3$ – (x/2) $Ca_2Fe_{4/3}W_{2/3}O_6$ solid solution.

Composition (x)	Calcining Temperature (°C)	Annealing Temperature (°C)
0 (BFO)	-	880
0.1-0.25	900	900
0.5-0.75	1000	1000
1 (CFWO)	900	1200

Table S2: Average compositions determined from 12 separate points by SEM-EDX for each intermediate composition in the series (1-x)$BiFeO_3$ – (x/2)$Ca_2Fe_{4/3}W_{2/3}O_6$.

Composition (x)	Target Composition	EDX Composition
0.1	$Ca_{0.2}Bi_{1.8}Fe_{1.9334}W_{0.0777}O_6$	$Ca_{0.182(52)}Bi_{1.82(37)}Fe_{1.9504(44)}W_{0.0495(56)}O_6$
0.15	$Ca_{0.3}Bi_{1.7}Fe_{1.9}W_{0.1}O_6$	$Ca_{0.228(49)}Bi_{1.77(39)}Fe_{1.889(23)}W_{0.111(19)}O_6$
0.2	$Ca_{0.4}Bi_{1.6}Fe_{1.8667}W_{0.133}O_6$	$Ca_{0.371(51)}Bi_{1.51(27)}Fe_{1.852(18)}W_{0.326(29)}O_6$
0.25	$Ca_{0.5}Bi_{1.5}Fe_{1.8335}W_{0.1775}O_6$	$Ca_{0.489(41)}Bi_{1.52(10)}Fe_{1.842(12)}W_{0.158(64)}O_6$
0.5	$CaBiFe_{1.667}W_{0.333}O_6$	$Ca_{1.078(61)}Bi_{0.922(43)}Fe_{1.667(27)}W_{0.369(26)}O_6$
0.75	$Ca_{1.5}Bi_{0.5}Fe_{1.5}W_{0.5}O_6$	$Ca_{1.504(13)}Bi_{0.496(44)}Fe_{1.476(13)}W_{0.524(34)}O_6$

Table S3: Structural refinement of $Ca_2Fe_{4/3}W_{2/3}O_6$ in *Pnma* from a combined Rietveld refinement on synchrotron x-ray diffraction data and TOF neutron diffraction data collected at Beamline I11, Diamond Light Source and POWGEN, ORNL respectively.

Atom	*x*	*y*	*z*	Occupancy	Site	B_{iso} (Å)
Space Group: *Pnma*, R_{wp} = 4.359 % *a* = 5.5235(3) Å, *b* = 7.7054(5) Å, *c* = 5.4172(4) Å, α = β = γ = 90°.						
Ca	0.4592(2)	0.25	-0.0109(6)	0.998(2)	4c	1.12(3)
Fe	0	0	0	0.667(2)	4a	0.507(4)
W				0.322(2)		
O1	0.2067(6)	0.0452(4)	0.7901(6)	1	8d	0.53(1)
O2	-0.0247(9)	0.25	0.0821(7)	1	4c	0.49(1)

Table S4: Extracted parameters from magnetic susceptibility measurements.

**Composition (x)**	**Néel Temperature (K)**	**Coercive Field (T)**	**Saturation Magnetisation (µB / Fe)**	R^2
CFWO (1)	355 (1)	0.235 (4)	0.00849 (4)	0.99989
0.75	465 (2)	0.798 (7)	0.0135 (1)	0.99986
0.5	565 (2)	0.810 (4)	0.01052 (5)	0.99987
0.25	599 (1)	1.899 (6)	0.00881 (3)	0.99993
0.2	651 (2)	1.424 (6)	0.00847 (5)	0.99988
0.15	621 (2)	1.079 (6)	0.00804 (6)	0.99985
0.1	624 (2)	0.723 (9)	0.00670 (9)	0.99944
BFO (0)	640	0	0	N/A

Figures S1: M(T) for intermediate compositions within the (1-x)BiFeO3 – (x/2) $Ca_2Fe_{4/3}W_{2/3}O_6$ solid solution. Due to the reducing atmosphere in the high temperature furnace, small amounts of Fe_3O_4 formed meaning that it was not possible to extract the effective magnetic moment by a Curie-Weiss fit.

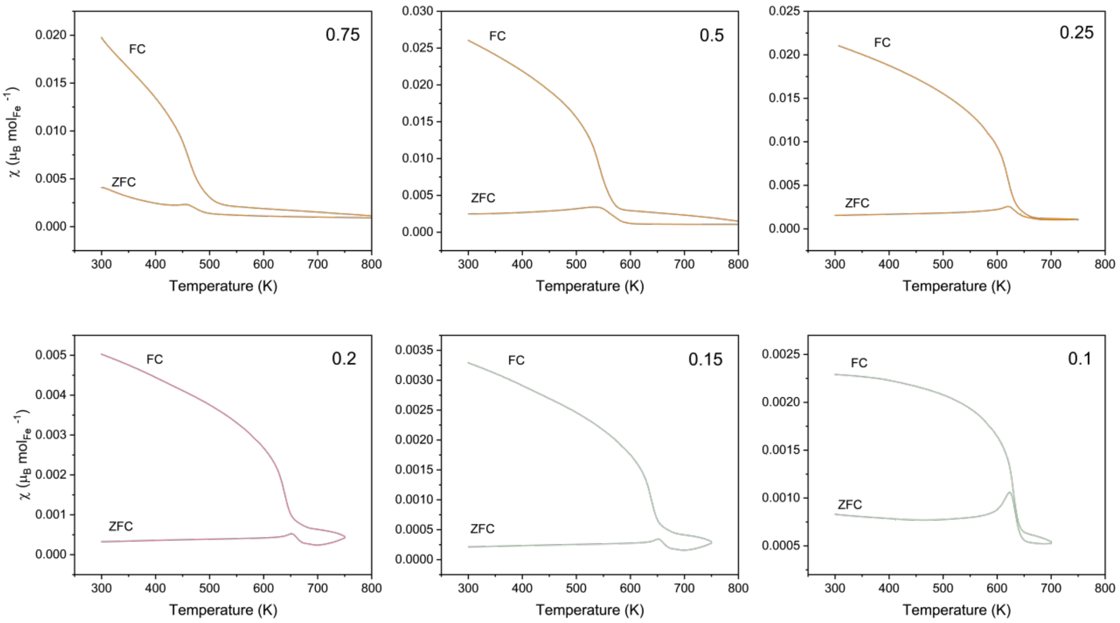

Figure S2: M(H) for (left) rhombohedral compositions and (right) orthorhombic compositions.

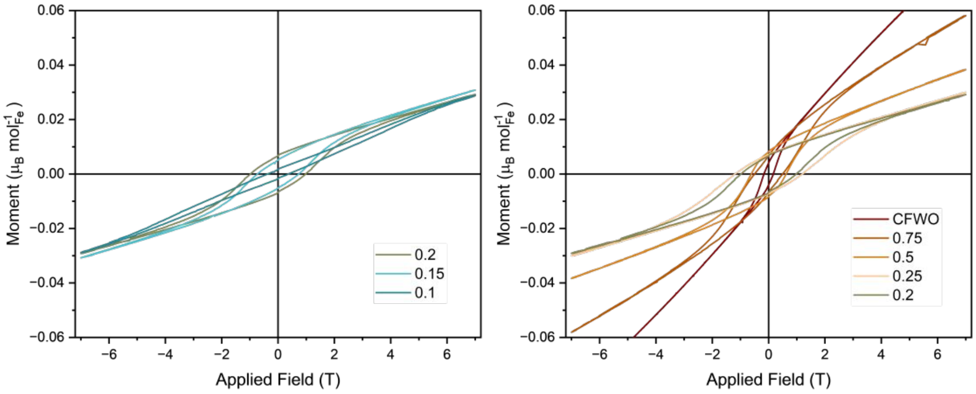

Table S5: The trialled magnetic subgroups and R_{wp} from a Rietveld refinement on PND data for a select composition from each change in magnetic symmetry. The magnetic moments were allowed to refine freely except from the weak FM canting which was constrained to zero.

**Orthorhombic**				**Rhombohedral**			
**x**	**Space group**	R_{wp} **(%)**	**Magnitude (μ_B per Fe)**	**x**	**Space group**	R_{wp} **(%)**	**Magnitude (μ_B per Fe)**
**0.75**	*Pnma*.1 (62.441)	4.694	3.07 (8)	**0.1**	*R*3*c*.1 (161.69)	6.499	3.87 (2)
	Pn′m′a (62.446)	4.445	3.21 (2)		*R*3*c′* (161.71)	14.724	0[1]
	Pnm′a′ (62.447)	8.726	0.5 (6)		*Cc*.1 (9.37)	5.514	3.85 (6)
	Pn′ma′ (62.448)	4.674	3.24 (3)		*Cc′* (9.39)	6.283	3.90 (2)
**0.5**	*Pnma*.1 (62.441)	6.311	3.79 (2)	**0.15**	*R*3*c*.1 (161.69)	8.718	3.95 (2)
	Pn′m′a (62.446)	5.176	3.98 (2)		*R*3*c′* (161.71)	15.617	0[1]
	Pnm′a′ (62.447)	11.876	0.6 (7)		*Cc*.1 (9.37)	6.721	4.28 (8)
	Pn′ma′ (62.448)	4.609	3.88 (2)		*Cc′* (9.39)	6.551	3.78 (2)

Figure S3: A combined Rietveld refinement of x = 0.75 on PXRD data collected at Beamline I11 (λ = 0.82586 (4) Å) at DLS (right) and NPD (left) data collected at D2B (λ = 1.5951 (1) Å) at the ILL.[1]values refined to ~0.00001 with errors that were 5 orders of magnitude larger.

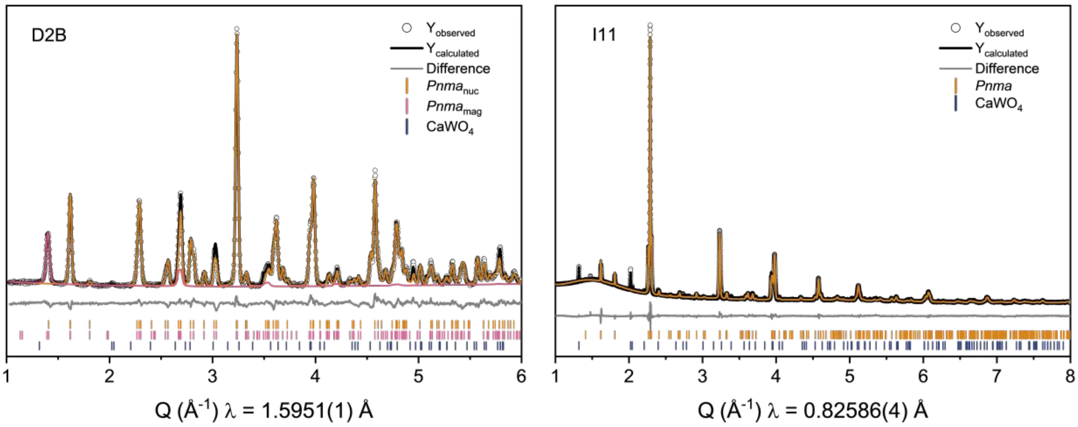
D2B
Y observed
Y calculated
Difference
Pnma nuc
Pnma mag
CaWO4
Q (Å-1) λ = 1.5951(1) Å
I11
Y observed
Y calculated
Difference
Pnma
CaWO4
Q (Å-1) λ = 0.82586(4) Å

Table S6: Structural details of the Orthorhombic (*Pnma*) phase of x = 0.75 extracted from a combined Rietveld refinement of PXRD and NPD data

Atom	*x*	*y*	*z*	Occupancy	Site	B_{iso} (Å)
Space Group: *Pnma*, R_{wp} = 3.847 % *a* = 5.5436(2) Å, *b* = 7.7614(2) Å, *c* = 5.4552(2) Å, α = β = γ = 90°.						
Bi	0.5447(3)	0.25	-0.0044(7)	0.262	4c	1.82(4)
Ca				0.752		
Fe	0	0	0	0.738	4a	0.43(2)
W				0.262		
O1	0.2931(3)	0.0407(2)	0.7931(3)	1	8d	0.97(3)
O2	-0.0219(4)	0.25	0.0772(4)	1	4c	0.90(4)

Figure S4: A combined Rietveld refinement of x = 0.5 on PXRD data collected at Beamline I11 (λ = 0.82586 (4) Å) at DLS (right) and NPD (left) data collected at D2B (λ = 1.5951 (1) Å) at the ILL.

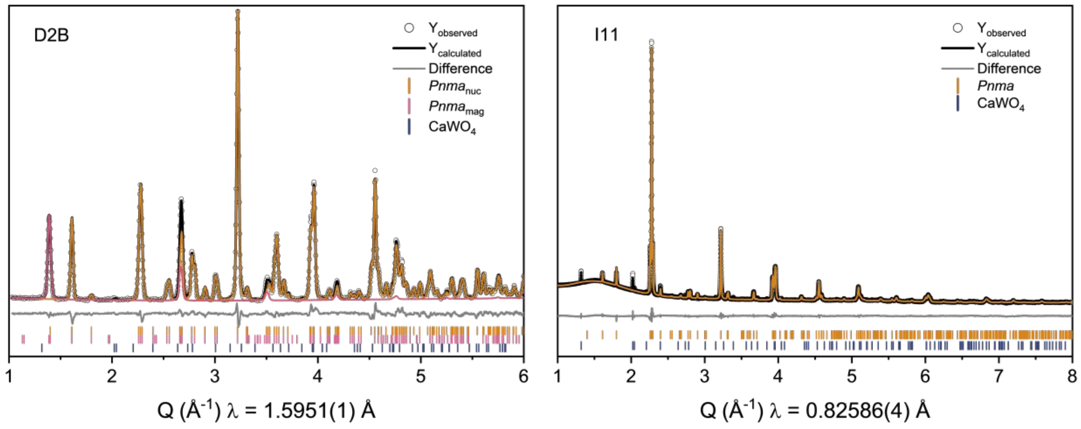

Table S7: Structural details of the Orthorhombic (*Pnma*) phase of x = 0.5 extracted from a combined Rietveld refinement of PXRD and NPD data

Atom	*x*	*y*	*z*	Occupancy	Site	B_{iso} (Å)
Space Group: *Pnma*, R_{wp} = 4.615 % *a* = 5.5635(5) Å, *b* = 7.80603(7) Å, *c* = 5.4809(5) Å, α = β = γ = 90°.						
Bi	0.5430(9)	0.25	-0.0021(4)	0.509	4c	2.20(2)
Ca				0.491		
Fe	0	0	0	0.870	4a	0.43(2)
W				0.130		
O1	0.2873(5)	0.0427(4)	0.7996(4)	1	8d	1.10(5)
O2	-0.0167(6)	0.25	0.0713(7)	1	4c	0.86(7)

Figure S5: A combined Rietveld refinement of x = 0.25 on PXRD data collected at Beamline I11 (λ = 0.82586 (4) Å) at DLS (right) and NPD (left) data collected at D2B (λ = 1.5951 (1) Å) at the ILL.

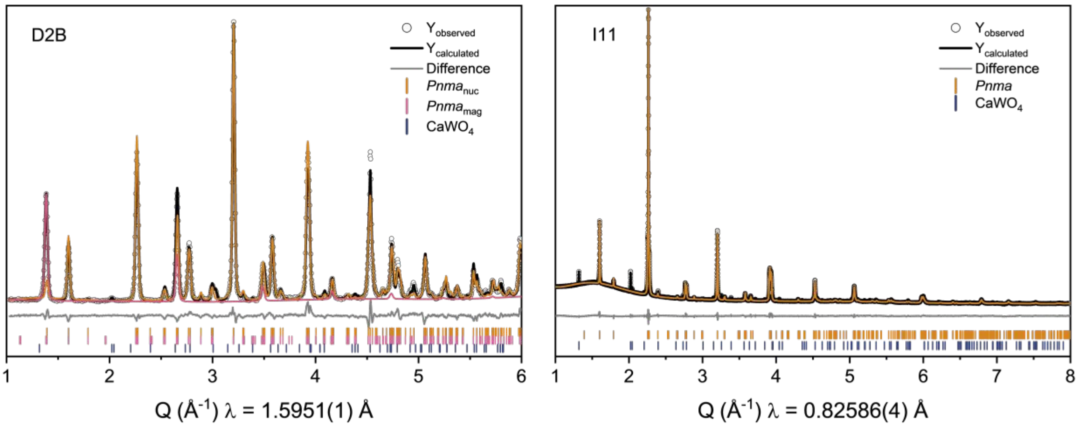

Table S8: Structural details of the Orthorhombic (*Pnma*) phase of x = 0.25 extracted from a combined Rietveld refinement of PXRD and NPD data

Atom	*x*	*y*	*z*	Occupancy	Site	B_{iso} (Å)
Space Group: *Pnma*, R_{wp} = 6.244 % *a* = 5.5713(3) Å, *b* = 7.8501(3) Å, *c* = 5.5219(3) Å, α = β = γ = 90°.						
Bi	0.5269(2)	0.25	-0.006(1)	0.752	4c	2.56(7)
Ca				0.248		
Fe	0	0	0	0.921	4a	0.32(4)
W				0.079		
O1	0.2863(7)	0.0428(5)	0.7869(7)	1	8d	2.32(7)
O2	-0.0162(9)	0.25	0.0703(10)	1	4c	1.33(7)

Figure S6: A combined Rietveld refinement of x = 0.15 on PXRD data collected at Beamline I11 (λ = 0.82586 (4) Å) at DLS (top) and NPD data collected at D2B (λ = 1.5951 (1) Å) at the ILL.

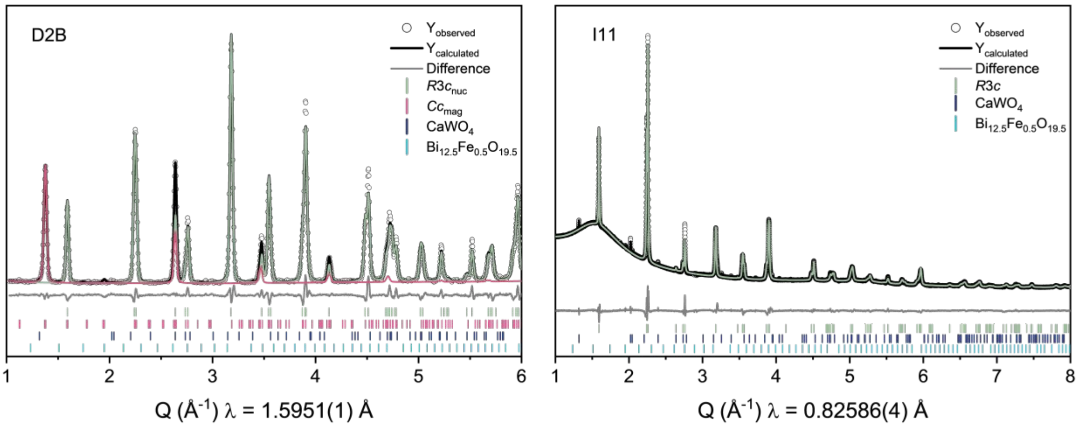

Table S9: Structural details of the rhombohedral ($R3c$) phase of x = 0.15 extracted from a combined Rietveld refinement of PXRD and NPD data

Atom	x	y	z	Occupancy	Site	B_{iso} (Å)
Space Group: $R3c$, R_{wp} = 6.216 % $a = b$ = 5.5700(6) Å, c = 13.787(1) Å, α = β = 90°, γ = 120°.						
Bi	0	0	0.8120(4)	0.886	6a	0.99(5)
Ca				0.114		
Fe	0	0	0.0369(4)	0.968	6a	0.59(3)
W				0.032		
O	0.6540(4)	0.7637(3)	0.1033(2)	1	18b	0.90(3)

Figure S7: A combined Rietveld refinement of x = 0.1 on PXRD data collected at Beamline I11 (λ = 0.82586 (4) Å) at DLS (right) and NPD (left) data collected at D2B (λ = 1.5951 (1) Å) at the ILL.

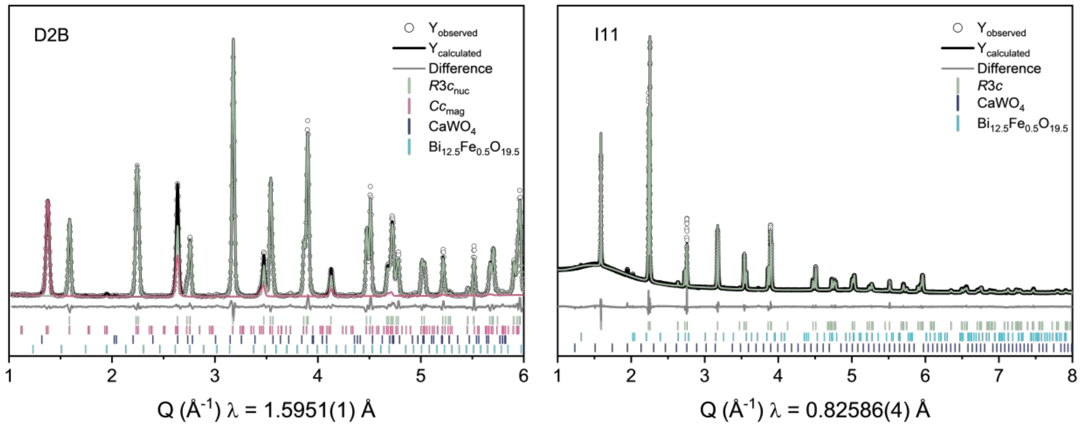

Table S10: Structural details of the rhombohedral ($R3c$) phase of x = 0.1 extracted from a combined Rietveld refinement of PXRD and NPD data

Atom	x	y	z	Occupancy	Site	B_{iso} (Å)
Space Group: $R3c$, R_{wp} = 5.4703 % $a = b$ = 5.5686(8) Å, c = 13.804(2) Å, α = β = 90°, γ = 120°.						
Bi	0	0	0.8125(6)	0.909	6a	0.95(5)
Ca				0.091		
Fe	0	0	0.0354(5)	0.975	6a	0.34(3)
W				0.025		
O	0.6512(3)	0.7611(2)	0.1012(1)	1	18b	0.78(2)

Figure S8: A combined Rietveld refinement of x = 0.2 on PXRD data collected at Beamline I11 (λ = 0.82586 (4) Å) at DLS (right) and NPD (left) data collected at D2B (λ = 1.5951 (1) Å) at the ILL.

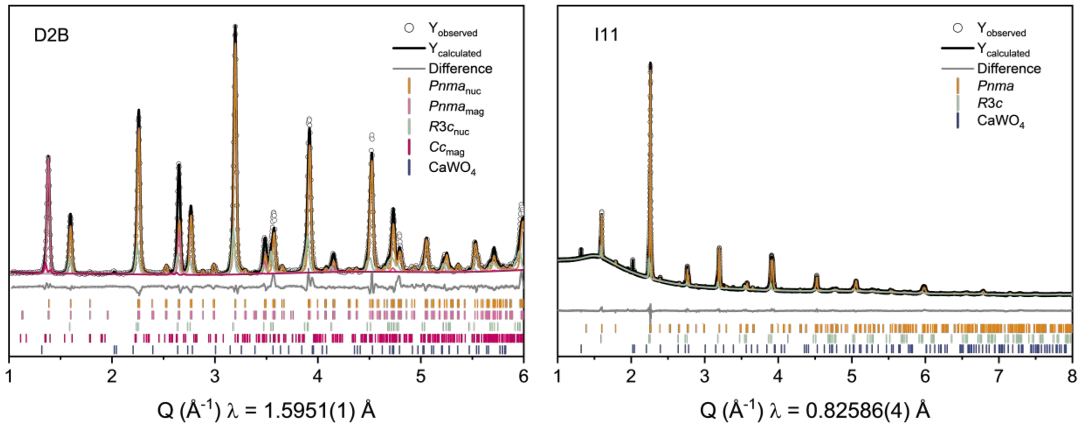

Table S11: Structural details of the rhombohedral (*R*3*c*) and orthorhombic (*Pnma*) phases of x = 0.2 extracted from a combined Rietveld refinement of PXRD and NPD data

**Atom**	***x***	***y***	***z***	**Occupancy**	**Site**	B_{iso} **(Å)**
Space Group: *Pnma*, R_{wp} = 5.870 % *a* = 5.5774(5) Å, *b* = 7.8611(8) Å, *c* = 5.5375(5) Å, α = β = γ = 90°.						
Bi	0.5166(1)	0.25	-0.006(1)	0.806	4c	2.81(1)
Ca				0.193		
Fe	0	0	0	0.930	4a	0.52(6)
W				0.070		
O1	0.2865(9)	0.0481(5)	0.7849(9)	1	8d	1.79(2)
O2	-0.0078(9)	0.25	0.0595(11)	1	4c	1.10(2)

**Atom**	***x***	***y***	***z***	**Occupancy**	**Site**	B_{iso} **(Å)**
Space Group: *R*3*c*, R_{wp} = 5.870 % *a* = *b* = 5.5654(8) Å, *c* = 13.777(2) Å, α = β = 90°, γ = 120°.						
Bi	0	0	0.8195(4)	0.806	6a	1.15(6)
Ca				0.193		
Fe	0	0	0.0383(3)	0.930	6a	0.34(3)
W				0.070		
O	0.653(1)	0.748(2)	0.1058(6)	1	18b	0.56(2)

Figure S9a: the compensated AFM canting along *a* and **b**. weak FM canting along *c* for x = 0.75. **c**. the compensated AFM canting along *a* and **d**. weak FM canting along *b* for x = 0.5.

.

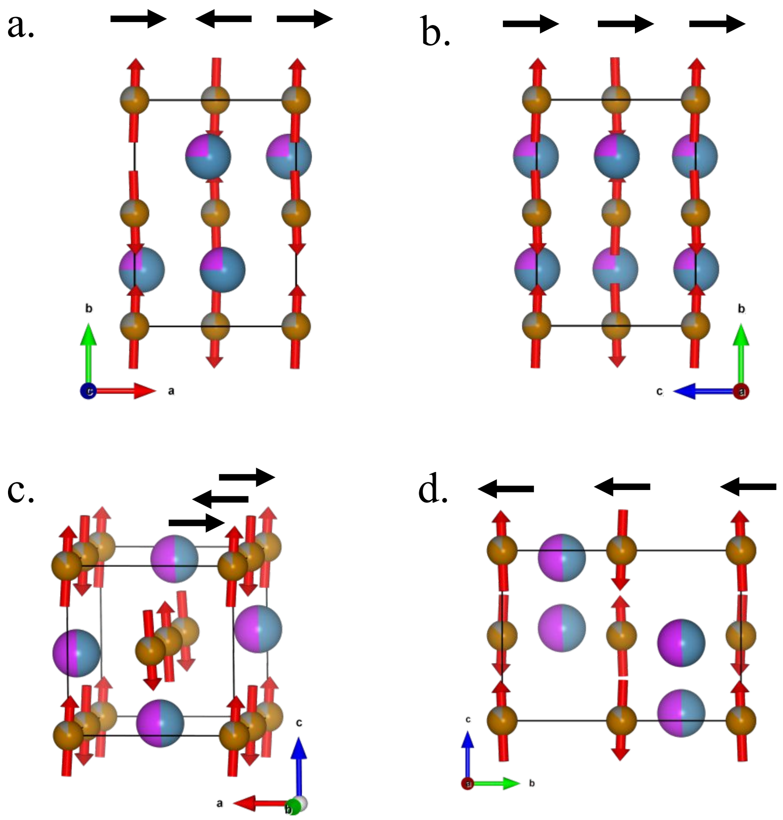

Figure S10: Rietveld fit of the most intense magnetic peak for x = 0.25 in magnetic space group 62.446 (left) and 62.448 (right). The R_{wp} for the models were 7.469 (62.446) and 5.378 (62.448).

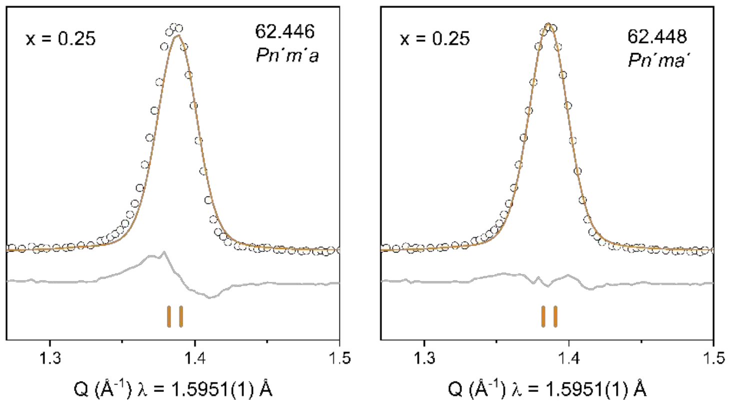

Figure S11: Rietveld output for x = 0.75 in 62.446 (a-d) and 62.448 (e-h) with variations in the canting of the magnetic moment modelled using fixed mode amplitudes generated using ISODISTORT. Visualisations of magnetic structures are provided below each refinement figure.

The models are as follows: in 62.446: **a**. freely refining mode amplitudes, **b**. increased AFM canting along *a*, **c**. increased FM canting along *c*, **d**. AFM with moments aligned along *a*. In 62.448: **e**. freely refining mode amplitudes, **f**. increased AFM canting perpendicular to *b*, **g**. increased FM canting along *b*, **h**. AFM with moments aligned along *a*.

The model in *Pn′m′a* (**S11a**) provides the best fit.

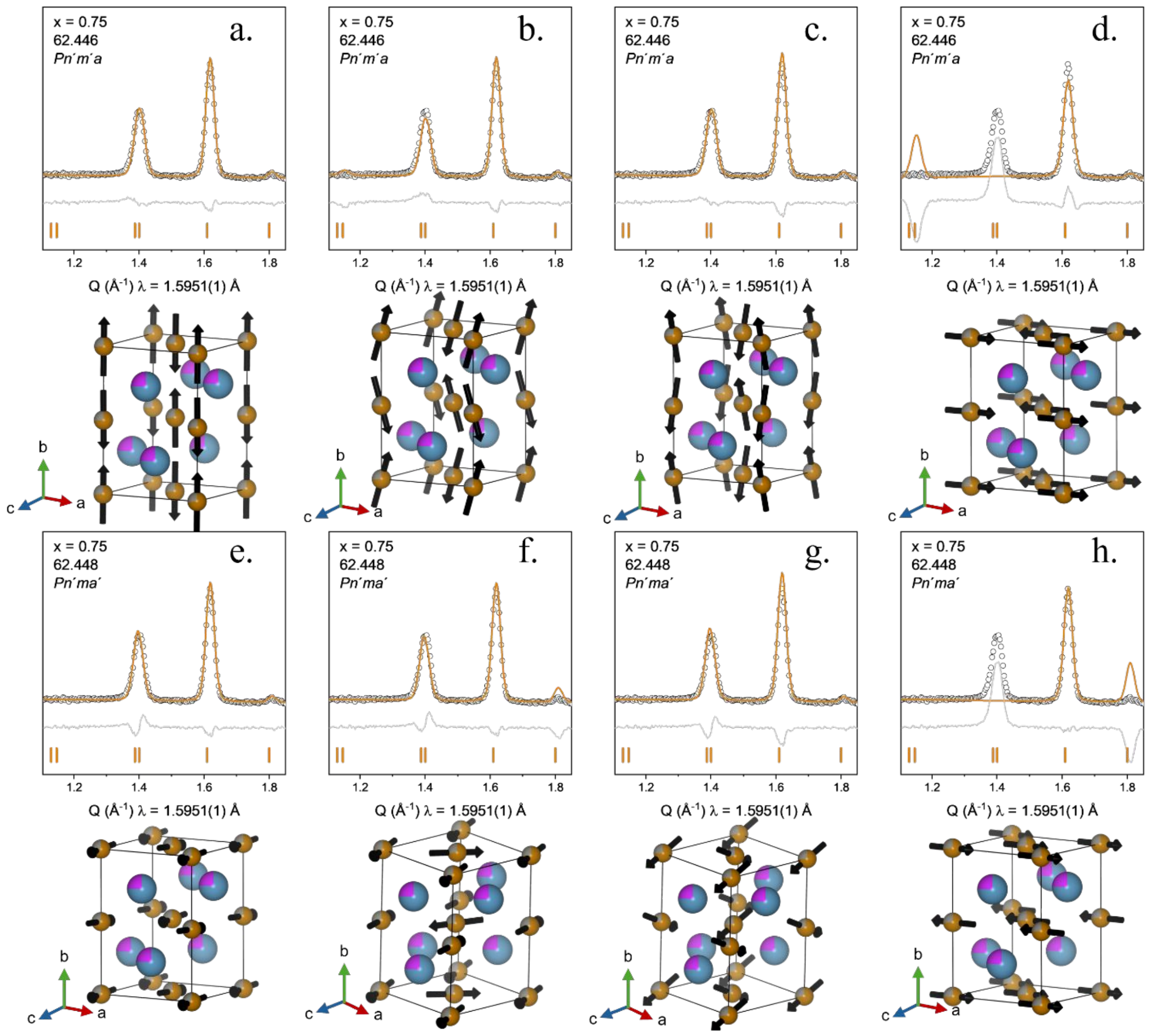

Figure S12: Rietveld output for x = 0.5 in 62.446 (a-d) and 62.448 (e-h) with variations in the canting of the magnetic moment modelled using fixed mode amplitudes generated using ISODISTORT. Visualisations of magnetic structures are provided below each refinement figure.

The models are as follows: in 62.446: **a**. freely refining mode amplitudes, **b**. increased AFM canting along *a*, **c**. increased FM canting along *c*, **d**. AFM with moments aligned along *a*. In 62.448: **e**. freely refining mode amplitudes, **f**. increased AFM canting perpendicular to *b*, **g**. increased FM canting along *b*, **h**. AFM with moments aligned along *a*.

The model in *Pn′ma′* (**S12e**) provides the best fit.

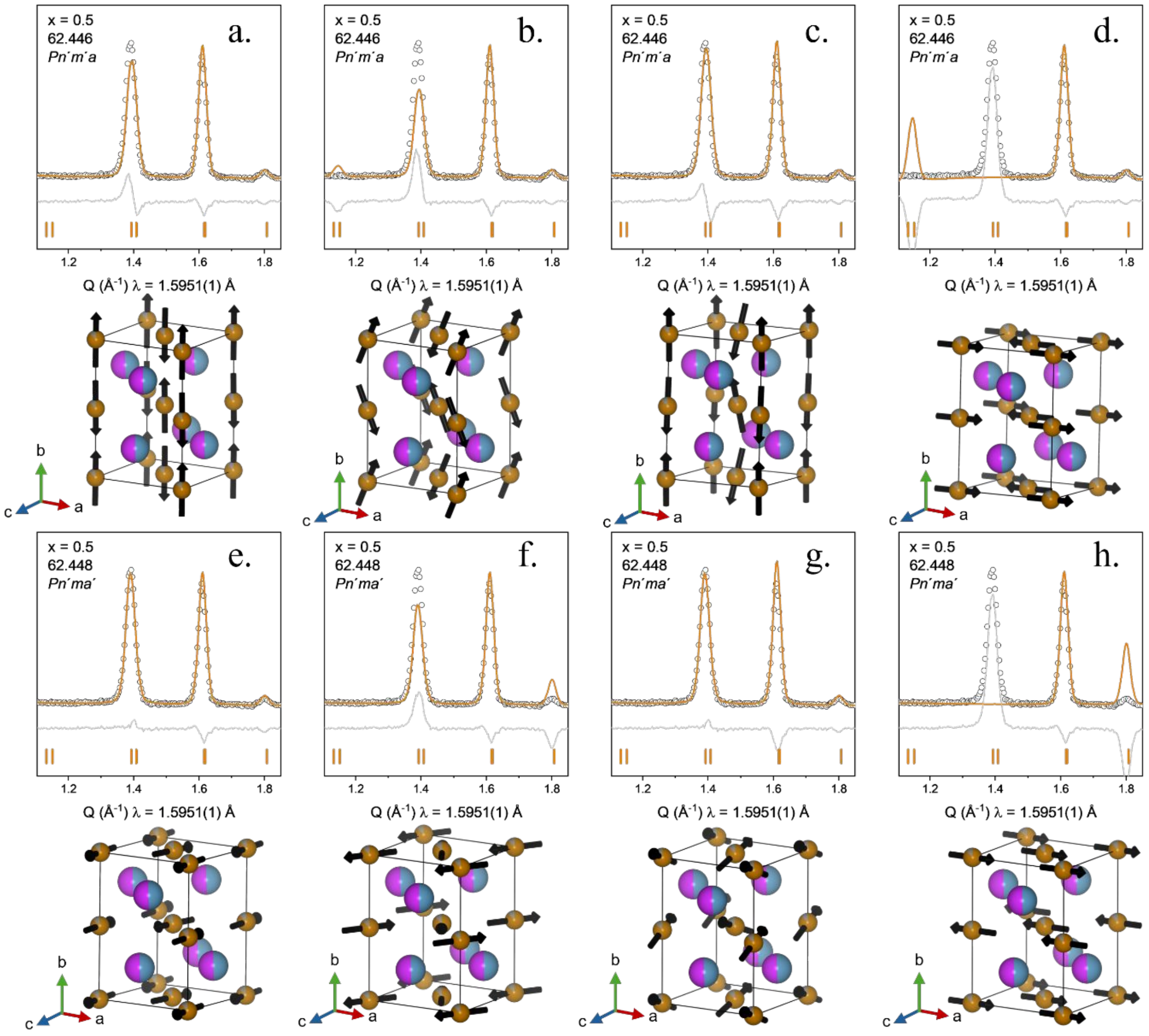

Figure S13: Rietveld output for x = 0.1 in 9.37 (a-d) and 9.39 (e,f) with variations in the canting of the magnetic moment modelled using fixed mode amplitudes generated using ISODISTORT. Visualisations of magnetic structures are provided below each refinement figure.

The models are as follows: in 9.37: **a**. freely refining mode amplitudes, **b**. increased FM canting along *b*, **c**. increased FM canting with freely refining contributions along *a* and *c*, **d**. AFM with moments aligned along *a*. In 9.39: **e**. freely refining mode amplitudes, **f**. increased FM canting perpendicular to *b*.

The model in *Cc*.1 (**S13a**) provides the best fit.

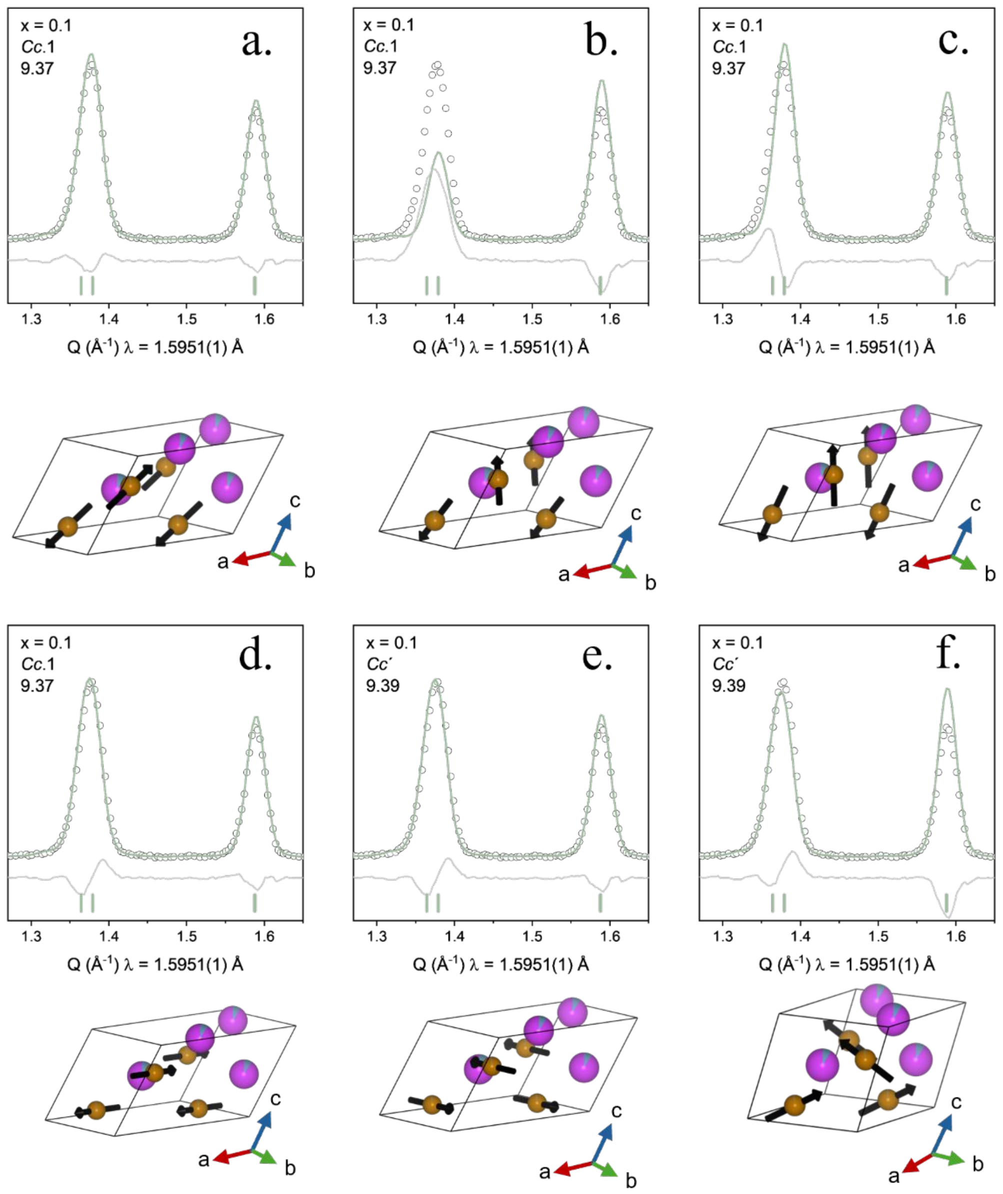

Figure S14: Rietveld output for x = 0.15 in 9.37 (a-d) and 9.39 (e,f) with variations in the canting of the magnetic moment modelled using fixed mode amplitudes generated using ISODISTORT. Visualisations of magnetic structures are provided below each refinement figure.

The models are as follows: in 9.37: **a**. freely refining mode amplitudes, **b**. increased FM canting along *b*, **c**. increased FM canting with freely refining contributions along *a* and *c*, **d**. AFM with moments aligned along *a*. In 9.39: **e**. freely refining mode amplitudes, **f**. increased FM canting perpendicular to *b*.

The model in *Cc*.1 (**S14a,d**) and *Cc*´ (**S14e**) all provide good fits, however the magnitude of the magnetic moment in the *Cc*.1 model are far larger than expected as discussed in the main text.

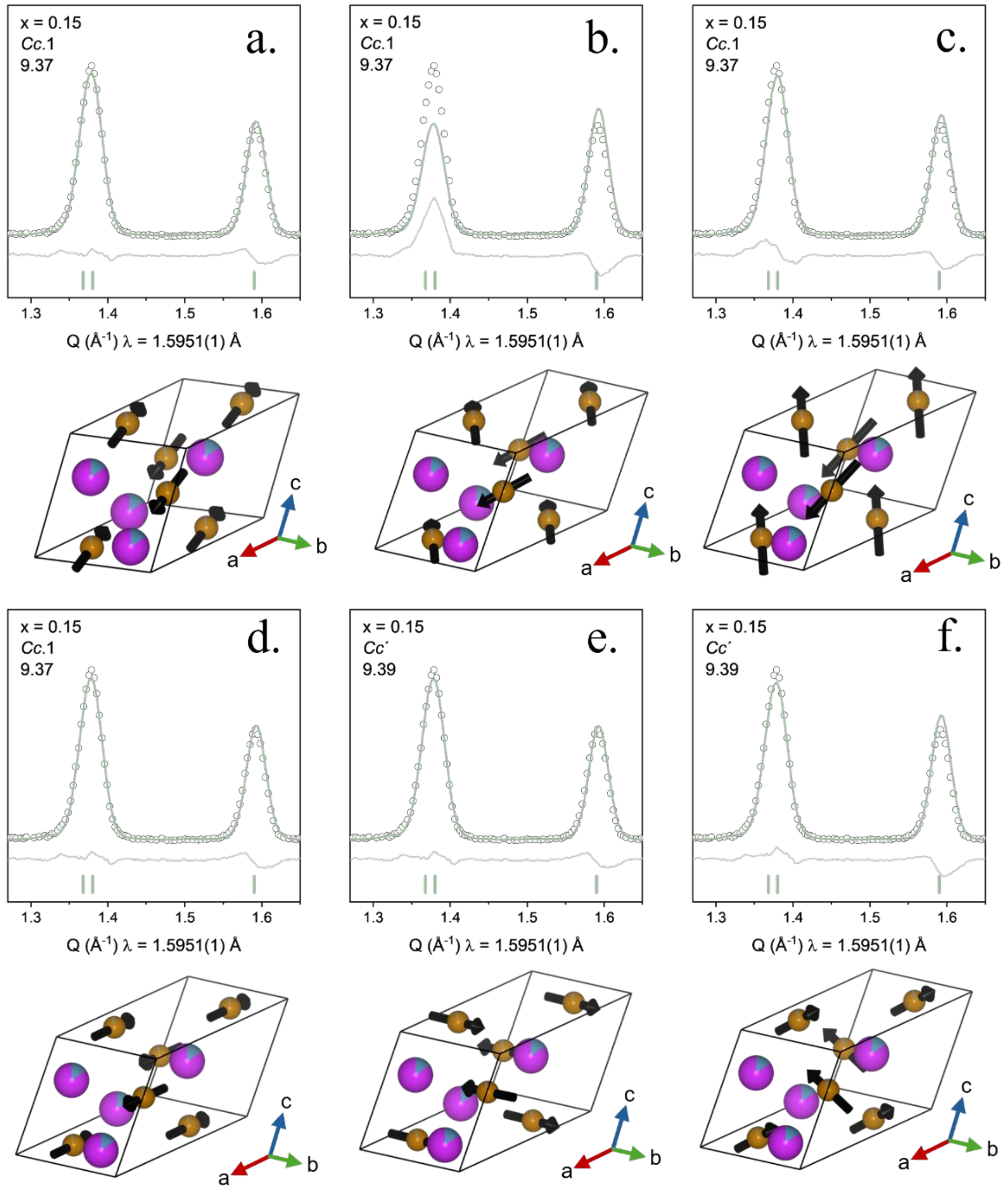

Supplement: Supplementary file 1 [file ic5c01516_si_001.pdf]
